# Autoimmune Response as a Mechanism for a Dobzhansky-Muller-Type Incompatibility Syndrome in Plants

**DOI:** 10.1371/journal.pbio.0050236

**Published:** 2007-09-04

**Authors:** Kirsten Bomblies, Janne Lempe, Petra Epple, Norman Warthmann, Christa Lanz, Jeffery L Dangl, Detlef Weigel

**Affiliations:** 1 Max Planck Institute for Developmental Biology, Tübingen, Germany; 2 Department of Biology, University of North Carolina, Chapel Hill, North Carolina, United States of America; 3 Department of Microbiology and Immunology, University of North Carolina, Chapel Hill, North Carolina, United States of America; 4 Carolina Center for Genome Sciences, University of North Carolina, Chapel Hill, North Carolina, United States of America; Oregon State University, United States of America

## Abstract

Epistatic interactions between genes are a major factor in evolution. Hybrid necrosis is an example of a deleterious phenotype caused by epistatic interactions that is observed in many intra- and interspecific plant hybrids. A large number of hybrid necrosis cases share phenotypic similarities, suggesting a common underlying mechanism across a wide range of plant species. Here, we report that approximately 2% of intraspecific crosses in Arabidopsis thaliana yield F_1_ progeny that express necrosis when grown under conditions typical of their natural habitats. We show that several independent cases result from epistatic interactions that trigger autoimmune-like responses. In at least one case, an allele of an *NB-LRR* disease resistance gene homolog is both necessary and sufficient for the induction of hybrid necrosis, when combined with a specific allele at a second locus. The A. thaliana cases provide insights into the molecular causes of hybrid necrosis, and serve as a model for further investigation of intra- and interspecific incompatibilities caused by a simple epistatic interaction. Moreover, our finding that plant immune-system genes are involved in hybrid necrosis suggests that selective pressures related to host–pathogen conflict might cause the evolution of gene flow barriers in plants.

## Author Summary

Hybridization brings together genetic material from different genomes. Sometimes, the novel combinations of genes are deleterious in the offspring, even though the genes were innocuous, or even beneficial, in their parents. Such “genetic incompatibilities” have been observed in crosses within and between species in plants, animals, and fungi, and could contribute to the maintenance of population or species boundaries. We have investigated a highly deleterious genetic incompatibility called hybrid necrosis that is observed in many plant taxa. Using different wild strains of Arabidopsis thaliana as a model*,* we show that hybrid necrosis is often associated with inappropriate activation of the plant immune system—effectively plant autoimmunity. We identified a gene in one strain that triggers necrosis when combined with a second locus from another strain. The product of this gene is an NB-LRR protein, the most common type of plant disease resistance protein. This finding raises the possibility that selective pressure exerted by pathogens can promote rapid evolution of gene variants that might provide benefits to the parent lineage but can cause serious problems for hybrid progeny.

## Abstract 3

Sometimes, genes that are innocuous in the parents are deleterious when combined in the offspring. Here, some genes involved in hybrid necrosis in plants have been identified.

## Introduction

Epistasis—the nonadditive interaction between genes—is a critical determinant for the performance of hybrid genotypes. It encompasses a spectrum of important genetic phenomena, from positive heterosis (hybrid vigor) to negative heterosis (hybrid sterility or lethality). In plants, two prevalent examples of the latter are cytoplasmic male sterility [1] and hybrid necrosis or weakness [2]. Cytoplasmic male sterility and hybrid necrosis have unfortunately received relatively little attention outside the plant genetics literature, even though one of the earliest described cases of hybrid necrosis is between two *Crepis* species and conforms to the tenets for Dobzhansky-Muller type incompatibilities, which establish or maintain gene flow barriers between species [3,4]. The Dobzhansky-Muller model posits that genetic incompatibility in hybrids results from deleterious interactions between alleles that, while innocuous in their native genomic context, have followed independent evolutionary trajectories in each parental lineage [3]. The Dobzhansky-Muller model is agnostic with respect to why causal genes diverge, but evidence from several studies in *Drosophila* suggests that hybrid incompatibility may arise as a by-product of adaptive evolution, since the few known incompatibility genes are all rapidly evolving [5–7]. However, despite advances in our knowledge of the genetics of hybrid incompatibility, the prevalence and mechanisms of early-arising gene flow barriers between members of the same species are not yet well understood.

Because hybrid necrosis is found in both inter- and intraspecific crosses, it may result from recurrent evolutionary processes observed at different temporal levels of genetic divergence [2]. Strikingly, hybrid necrosis cases, whether observed in out-crossing or inbreeding plants, share a suite of phenotypic properties that suggest a common underlying mechanism. Among these is that they are generally genetically simple, with often only two causal loci involved.

We investigated whether the reference plant Arabidopsis thaliana might serve as a model for the mechanistic understanding of hybrid necrosis. Surprisingly, hybrid necrosis in crosses between A. thaliana strains occurs with a prevalence of about 2% (among 861 unique F_1_ progenies derived from crosses between 280 distinct parental strains). The 20 cases of hybrid necrosis that we identified are caused by at least five genetically independent systems, each involving epistatic interactions of two to four loci. We demonstrate that four independent cases involve activation of genes typically associated with plant response to pathogen attack, suggesting that autoimmune-like responses are triggered by different epistatic interactions in these hybrids. We demonstrate for one case that this response, and the consequent hybrid necrosis, requires an *NB-LRR* disease resistance gene homolog from one parent, which interacts with an allele at a single locus from a second strain. The involvement of the plant immune system in several independent cases of hybrid necrosis implicates aberrant activation of disease resistance signaling as a general mechanism. The phenotypic similarities of hybrid necrosis cases in other species to those we describe in *A. thaliana,* including in the expression of defense-related genes [8,9], suggest that A. thaliana is an accessible model for studying the molecular causes of this type of genetic incompatibility [2]. Since hybrid necrosis afflicts interspecific hybrids in several taxa, it is conceivable that in some cases hybrid necrosis may give rise to gene flow barriers. As a working hypothesis, we propose that host–pathogen conflict could drive a recurrent genetic syndrome that might contribute to the establishment or maintenance of certain hybrid incompatibilities in plants.

## Results

### Mapping of Two Incompatible Genomic Regions

**Figure 1:**
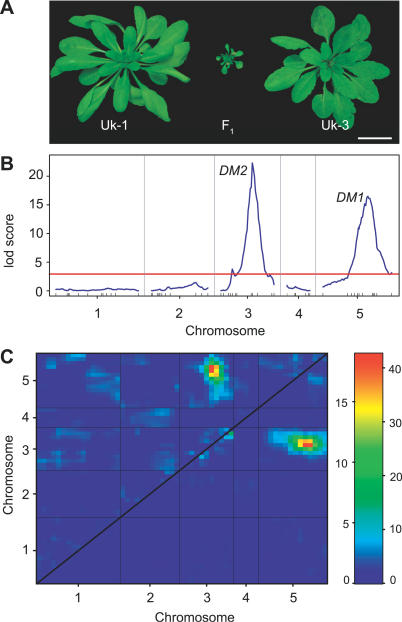
Uk-1 × Uk-3 Hybrid Phenotype and QTL Mapping (A) Uk-1 (left), Uk-1/Uk-3 F_1_ hybrid (middle), and Uk-3 (right) plants grown at 16 °C. Scale bar represents 2 cm. (B) QTL mapping using 181 individuals identifies two regions associated with the F_1_-like phenotype in F_2_ populations. Red line delineates significance threshold (*p* = 0.05, established by 1,000 permutations); tick marks indicate marker positions. (C) Heat map for two-dimensional genome scan with a two-QTL model for maximum joint lod (upper triangle) and interaction lod (lower triangle) scores. Color-coded scale indicates values for epistatic interaction on the left, and joint lod score on the right.

Our foray into genetic incompatibilities started with the observation that the F_1_ progeny from a cross of A. thaliana strains Uk-1 and Uk-3 became severely necrotic and did not reach the flowering stage (Figure 1A). The phenotype of the Uk-1/Uk-3 hybrids resembled previously described cases of hybrid necrosis in other plants, which similarly involve signs of oxidative stress such as leaf lesioning, yellowing, and abnormal morphologies [2]. The compromised phenotype was fully penetrant at 16 °C, a habitat temperature typical for A. thaliana in the wild [10], but could be conveniently suppressed at 23 °C, allowing seeds to be obtained for genetic analysis. The “F_1_” phenotype reappeared at high frequency in the F_2_ generation, together with a more severe growth arrest phenotype, suggesting a relatively simple genetic basis (Table 1).

**Table 1:**
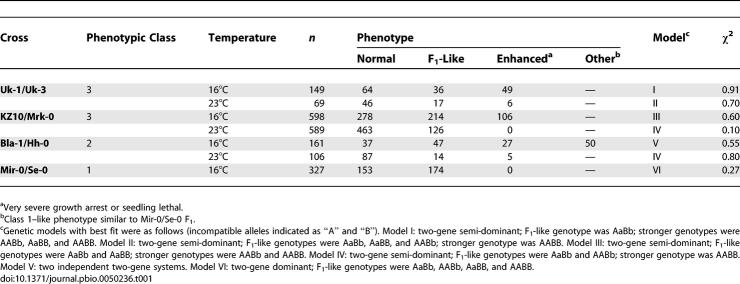
F_2_ Segregation Ratios

We investigated how many genomic regions contribute to the necrotic phenotype by performing whole-genome single nucleotide polymorphism (SNP) scans of F_2_ plants that exhibited the F_1_-like phenotype. Although there was some ambiguity in classifying plants as belonging to either the “F_1_” or the more severe “F_2_” phenotypic class, quantitative trait locus (QTL) mapping identified two unlinked regions, one contributed by each parent, as being the major loci responsible for hybrid necrosis. The *DM1 (DANGEROUS MIX 1)* locus was derived from Uk-3 and mapped to Chromosome 5, while the *DM2* locus from Uk-1 mapped to Chromosome 3 (Figure 1B). Lines with each region introgressed separately into the common laboratory accession, Col-0, were phenotypically indistinguishable from pure Col-0. However, crosses between the Chromosome 3 (Uk-1) and Chromosome 5 (Uk-3) introgression lines yielded F_1_ plants that recapitulated the Uk-1/Uk-3 necrotic phenotype (not shown). Thus, we conclude that the epistatic interaction between the derived alleles of *DM1* from Uk-3 and *DM2* from Uk-1 is both necessary and sufficient for necrosis of Uk-1/Uk-3 hybrids. This finding is consistent with the results from a two-dimensional genome scan under a two-QTL model (Figure 1C).

We selected 1,200 F_2_ plants with an “F_1_” phenotype for fine mapping, assuming that these were heterozygous for the *DM1* and *DM2* loci. We mapped *DM1* to a 43.2-kb interval in reference to the genome sequence from Col-0. The *DM1* region contains seven annotated genes in Col-0, including two tandem duplicate genes, *AT5G41740* and *AT5G41750,* that encode closely related Toll interleukin receptor (TIR)–type NB-LRR proteins. *NB-LRR* genes are the most common class of disease resistance *(R)* genes in plants [11]. An ethyl-methanesulfonate-induced missense mutation in the NB region has been described for an *AT5G41740/AT5G41750* allele in the No-0 accession. This mutation, *SSI4 (suppressor of salicylic acid insensitivity of npr1 4),* causes spontaneous lesioning and dwarfed growth associated with ectopic activation of plant immune responses at 23 °C [12]. We found that *SSI4* was seedling lethal at 16 °C. The similar necrotic phenotypes and similar temperature responsiveness of *ssi4* and the Uk-1/Uk-3 hybrid plants suggested that the Uk-3 allele of *AT5G41740/AT5G41750* was a candidate for *DM1*.

Because of difficulties with obtaining fine-scale recombinants of the correct phenotypic class, we were not able to narrow the final *DM2* interval to less than ∼148 kb (in the Col-0 reference genome sequence). The nonrecombining region spans *AT3G44550* to *AT3G44730,* which includes two annotated *NB-LRR* genes in Col-0 *(AT3G44630* and *AT3G44670)*.

**Figure 2:**
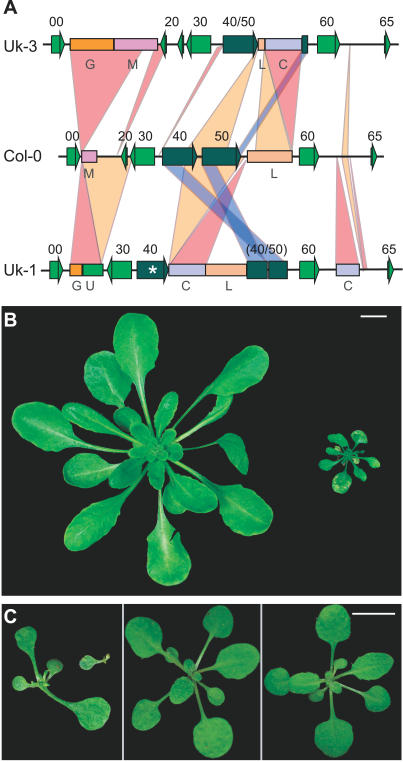
Identification of *DM1* as an *AT5G41740*/*AT5G41750* Allele (A) Schematic alignment of mapping interval. Genes are indicated with green boxes. Insertions relative to Col-0 are indicated with red triangles, deletions in beige, and duplications in blue. The last two digits of *AT5G417XX* gene identifiers are given above each line. *AT5G41740* and *AT5G41750* are *NB-LRR* genes. Uk-3 has only a single *AT5G41740*/*AT5G41750* gene that is divergent from both Col-0 genes. Parentheses for Uk-1 indicate that these open reading frames are incomplete; asterisk indicates stop codon. Transposons are labeled below each line: MULE (M), copia (C), LINE (L), gypsy (G), and gypsy-associated *UlpI* protease sequence (U). (B) Uk-1/Uk-3 F_1_ hybrids grown at 16 °C, with transgenic sibling constitutively expressing amiRNA targeting *AT5G41740*/*AT5G41750* on the left. (C) T_1_ plants carrying a Uk-3 genomic fragment that includes the *AT5G41740*/*AT5G41750* allele. From left to right: Uk-1, Col-0, and Uk-3. Scale bars in (B) and (C) represent 1 cm.

*NB-LRR* genes and *NB-LRR* gene clusters are often very polymorphic, varying both in sequence and gene copy number [13,14]. We therefore isolated and sequenced fosmids covering the *DM1* region from both Uk-1 and Uk-3. The 43.2-kb mapping interval in Col-0 corresponds to 46.2 kb in Uk-3, and 49.0 kb in Uk-1 (Figure 2A). There are numerous large-scale differences among the three accessions, many involving LINE, MULE, gypsy, and copia retrotransposons. The *NB-LRR* genes are also very different. Uk-1, which carries an innocuous haplotype at this locus, has two gene fragments that encode only the TIR and NB domains, as well as a full-length gene with a premature stop codon in its open reading frame. In contrast, Uk-3, which harbors the Uk-1-incompatible allele, contains only a single *NB-LRR* gene, which has an intact reading frame (Figure S1). The Uk-3 allele of this protein has 20% and 13.5% amino acid substitutions relative to AT5G41740 and AT5G41750, respectively.

### Hybrid Necrosis Caused by a Naturally Occurring *NB-LRR* Allele

We attempted to inactivate five candidate genes in the *DM1* region with artificial microRNAs (amiRNAs) [15]. amiRNA constructs were transformed into Uk-3, and first-generation transgenic plants (T_1_) were crossed with Uk-1. We recovered phenotypically normal Uk-1/Uk-3 F_1_ plants only when the plants carried amiRNAs targeting the *NB-LRR* gene (Figure 2B). Rescue was quantitative and varied depending on the T_1_ line used, but several transgenic lines segregated phenotypically normal plants that did not show any indication of necrosis at 16 °C. The suppression of hybrid necrosis co-segregated with the amiRNA transgene. Hence, the Uk-3 allele that corresponds to the *AT5G41740/AT5G41750* genes in Col-0 is necessary for hybrid necrosis in crosses with Uk-1.

To investigate whether the Uk-3 *NB-LRR* gene is also sufficient for the deleterious epistatic interaction, we transformed a 7.5-kb genomic fragment containing this gene into Uk-1, Uk-3, and Col-0. All eight independent T_1_ lines in Uk-1 exhibited a strong growth arrest phenotype even at 23 °C; this was not observed in any of 12 T_1_ lines in Col-0 or eight T_1_ lines in Uk-3 (Figure 2C). We also crossed Col-0 lines carrying the Uk-3 *NB-LRR* gene with Uk-1. F_1_ progeny exhibited strong hybrid necrosis indistinguishable from that observed in F_1_ plants obtained in Uk-1 × Uk-3 crosses, confirming that no other Uk-3-specific region is required for the Uk-1/Uk-3 incompatibility. Together, these experiments identify an allele of an *NB-LRR* disease resistance gene homolog as *DM1*. The Uk-3 allele of *DM1* is both necessary and sufficient for hybrid necrosis in combination with the Uk-1 allele of *DM2,* which maps to an unlinked region on Chromosome 3.

The Uk-3 strain has been maintained in the laboratory since 1954 [16]. To address whether the Uk-3 *DM1* allele arose naturally, we test-crossed 46 accessions selected from the worldwide range of A. thaliana to Uk-1. Only one cross, to Nc-1, produced progeny with a phenotype very similar to the Uk-1/Uk-3 hybrid (not shown). To confirm genetically that the same locus is likely to be involved, we crossed Uk-3/Nc-1 F_1_ plants, which are phenotypically normal, with Uk-1. All progeny showed the incompatibility phenotype, indicating that the Uk-1-incompatible locus from Nc-1 resides at the same genomic location as that from Uk-3. This conclusion is further supported by the observation that markers closely linked to *DM1* in Nc-1 are identical in sequence to those in Uk-3. Nc-1 was originally collected near Nancy, France, which lies about 160 km from Umkirch, Germany, where Uk-3 originated. We also collected new strains in and around Umkirch itself, and found that one of them, Uk-6, is Uk-1-incompatible as well, and also features identical sequences at *DM1*-linked markers.

Though Uk-3, Uk-6, and Nc-1 carry similar haplotypes at *DM1,* all three strains are unique and diverged throughout the rest of the genome. Among 311 genome-wide SNPs, Nc-1 differs from Uk-3 at 132 positions, and Uk-6 differs from Uk-3 at 122 positions. This divergence is similar to differentiation between Uk-3 and numerous other accessions from the worldwide range of A. thaliana (which have from 102 to 188 differences at these 311 SNPs). Hence, the functionally relevant *DM1* polymorphism is not associated exclusively with a single, self-fertilizing lineage. Together, these data suggest that the *DM1* allele originally identified in Uk-3 arose, and has been maintained, in the wild.

### Genetic Incompatibility Due to Autoimmunity

**Figure 3:**
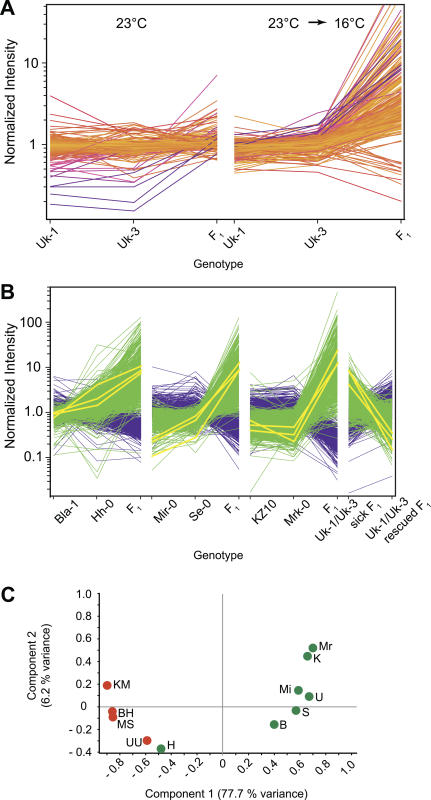
Microarray Analysis of Necrotic Hybrids (A) Expression profiles of Uk-1, Uk-3, and F_1_ hybrids. Genes shown differ significantly between the F_1_ and the two parents at 16 °C, but do not differ between the two parents. (B) Profiles of 1,080 genes that are significantly changed in at least one hybrid relative to its parents at 16 °C. Genes induced in hybrids are in green; suppressed genes are in blue. Yellow lines indicate three genes commonly used as markers of pathogen response (*PR1, PR2,* and *PR5;* see also Figure S3). In addition to profiles for three hybrids and their parents, regular Uk-1/Uk-3 hybrids (“sick”) are compared with siblings that are phenotypically rescued by a transgene that inactivates *DM1*. (C) PCA using the same set of 1,080 genes, with hybrids marked red and parents green. B, Bla-1; BH, Bla-1/Hh-0 F_1_; H, Hh-0; K, KZ10; KM, KZ10/Mrk-0 F_1_; Mi, Mir-0; Mr, Mrk-0; MS, Mir-0/Se-0 F_1_; S, Se-0; U, rescued Uk-1/Uk-3 F_1_; UU, nontransgenic Uk-1/Uk-3 F_1_.

The identification of *DM1* as an *NB-LRR* allele, together with the known effects of the *ssi4* mutation [12], suggested an autoimmune-like response as the mechanism of hybrid necrosis. To test this hypothesis, we turned to whole-genome transcriptome analyses. Because of the hybrid growth defects, we initially analyzed shoot apices including young leaves from plants that were raised at the permissive temperature of 23 °C, and then shifted to 16 °C. A few genes were differentially expressed at 23 °C, while many more genes were aberrantly expressed in the F_1_ plants after transfer to 16 °C (Figure 3A). We examined Gene Ontology annotations of genes differentially expressed at 16 °C, and all significantly overrepresented Gene Ontology categories were associated with immune system responses to infection (Table S1). This suggests that Uk-1/Uk-3 progeny suffer from an autoimmune response that is largely suppressed at 23 °C, but deleterious or lethal at 16 °C. The aberrant activation of immune responses in the F_1_ hybrids at 16 °C explains both the lethal phenotype and its sensitivity to environmental conditions. Constitutive immune response activation can cause dwarfing and autonecrosis similar to that observed in these hybrids (e.g., [17]), which, in some cases, is suppressed by higher temperature (e.g., [18,19]).

We also found that the amiRNAs against *DM1* altered the global expression profile of Uk1/Uk-3 hybrids, indicating down-regulation of autoimmune responses (Figure 3B, right panel). Thus, rescue at the phenotypic level was accompanied by rescue at the molecular level, further confirming the mechanistic association between the gene expression profiles and the hybrid necrosis phenotype triggered by interaction of the Uk-3 *DM1* allele with the *DM2* allele of Uk-1.

### Prevalence of Hybrid Necrosis and Hybrid Sterility in A. thaliana

To determine the frequency with which hybrid necrosis or other negative epistatic interactions occur in F_1_ progeny among A. thaliana strains, we performed crosses among a larger panel of wild strains and screened their F_1_ progeny for deleterious phenotypes (Tables S3 and S4). Together with the Uk crosses described above, this screen included 280 wild strains in 861 unique combinations. As before, we examined the viability and fertility of F_1_ hybrids at 16 °C, to mimic more closely the environment typical for the places of origin of A. thaliana accessions [10], rather than the standard laboratory growth temperatures of 20–23 °C. Based on phenotypes observed previously in inter-and intraspecific hybrids in other plants, we expected not only hybrid necrosis, but also early embryo (seed) lethality, sterility, or prezygotic barriers that prevent fertilization. No significant prezygotic barriers were observed; all crosses were successful and resulted in seed set. Fifteen crosses showed high seed abortion, both in the cross itself, and in the F_1_ progeny, but all of these could be attributed to known [20] or subsequently identified ploidy differences between the parents, and were not pursued further.

The majority of F_1_ hybrids were morphologically normal and vigorous at 16 °C, and, with the exception of the triploid progeny of the aforementioned interploidy crosses, none suffered from substantial (greater than about 20%) reduction in seed set, indicating normal or near-normal fertility. However, 20, or about 2% of combinations, developed a common set of symptoms, similar to those seen in Uk-1/Uk-3 hybrids (Table S4). Intercrosses between parents of necrotic hybrids indicated that the incompatibilities were attributable to at least five genetically distinct systems of epistatic interactions (Table S5).

**Figure 4:**
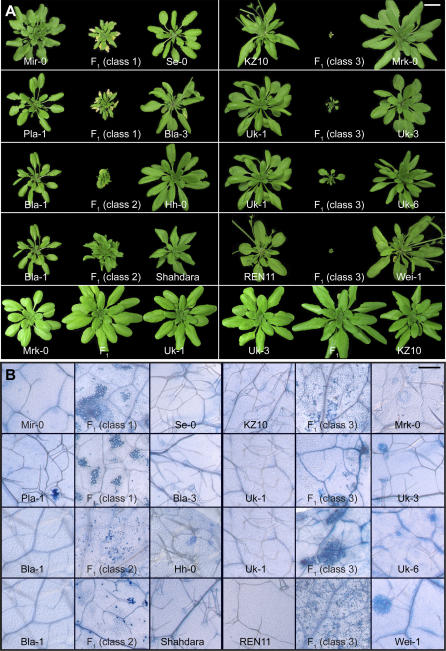
Range of Necrosis Phenotypes in A. thaliana Hybrids (A) Examples of necrotic hybrids representing three phenotypic classes defined in the text; 6-wk-old vegetative rosettes are shown. Two compatible hybrids, aged 5 wk, are shown in the bottom row for comparison. Scale bar (top right) represents 3 cm. (B) Trypan Blue staining for dead cells in leaves of hybrids and parents. Scale bar (top right) represents 250 μm.

Several phenotypes recurred in independent crosses (Figure 4A), including dwarfism, leaf abnormalities, lesioning, and extensive tissue necrosis similar to those seen in Uk-1/Uk-3 hybrids. These characteristics are also diagnostic of hybrid necrosis in other plants [2].

All hybrids tested were entirely or mostly rescued when plants were grown at 23 °C, like the Uk-1/Uk-3 hybrids. Importantly, several cases of hybrid necrosis in other plants were reported to be temperature sensitive [21–25]. These parallels strengthen the assertion that the examples of hybrid necrosis we have identified in A. thaliana share a common mechanism with, and thus provide a tractable model for, cases observed in other plants.

We grouped the 20 A. thaliana cases into three phenotypic categories (Figure 4A). Six hybrids (class 1) developed initially normally, but progressively succumbed to late-onset growth inhibition accompanied by extensive necrotic lesioning and cell death. Though they remained considerably smaller than their parents, these hybrids were still fertile at 16 °C. Eight hybrids (class 2) had more severe morphological defects compared to class 1 hybrids, including leaf warping, twisting, moderate to severe dwarfing, and loss of apical dominance. Macroscopically visible necrotic lesions were less pronounced than in class 1 hybrids, but there was nevertheless extensive cell death in leaves. Inflorescences were greatly reduced but still fertile at 16 °C. The six strongest cases (class 3, including Uk-1/Uk-3) were all severely stunted, with small, abnormal leaves that had a waterlogged appearance, and developed macroscopically visible lesions. These hybrids almost never flowered; if they did, they were sterile due to severe inflorescence and flower defects. REN11/Wei-1 hybrids were an exception; these sometimes produced tiny inflorescences at 16 °C, which in rare cases yielded a very small number of seeds.

In six tested cases (KZ10/Mrk-0, Uk-1/Uk-3, REN11/Wei-1, Bla-1/Hh-0, Mir-0/Se-0, and Bla-1/Shahdara), reciprocal crosses resulted in the same F_1_ hybrid necrosis phenotypes regardless of the direction of the cross, demonstrating that these are not caused by aberrant nuclear–cytoplasmic interactions, such as those involved in cytoplasmic male sterility [1].

### Autoimmunity among Independent Necrotic Hybrids

To investigate whether temperature-sensitive autoimmune-like responses similar to those observed in Uk-1/Uk-3 affected other hybrids, we performed microarray analysis on vegetative rosettes of plants either shifted from 23 °C to 16 °C (KZ10/Mrk-0) or grown continuously at 16 °C (Bla-1/Hh-0 and Mir-0/Se-0). In total, 1,080 genes were selected as differentially expressed in at least one F_1_ hybrid versus its parents, but not between the respective parents. As with the Uk1/Uk-3 hybrids, gene classes associated with immune responses [26] were overrepresented in this larger list as well (Table S2). This included many well-known genes commonly used as molecular markers, or genetically required for induction of pathogen response, such as *NDR1 (NON-RACE SPECIFIC RESISTANCE 1), EDS1 (ENHANCED DISEASE SUSCEPTIBILITY 1), PAD4 (PHYTOALEXIN DEFICIENT 4), PR1 (PATHOGENESIS RELATED 1), BGL2* (*BETA-1,3-GLUCANASE 2*)/*PR2,* and *PR5,* which were strongly induced in all hybrids compared with their parents (Figures 3B and S3).

The 1,080 differentially expressed genes exhibited strikingly similar expression profiles across all of the hybrid combinations (Figure 3B), which was readily apparent in a principal component analysis (PCA; Figure 3C). PCA clustered the F_1_ hybrids together, widely separated from all parental accessions, with one exception, Hh-0. That Hh-0 stands out is consistent with many of the 1,080 genes being expressed differently at 16 °C in Hh-0 compared to the other parents (Figure 3B). The Bla-1/Hh-0 F_1_ hybrid thus appears to exacerbate a subthreshold, constitutive immune response present in the Hh-0 parent; there is no evidence for such an effect in other genotypic combinations.

A common consequence of plant immune responses to infection is the hypersensitive response (HR), which can contribute to halting the spread of pathogens that require living tissue [27]. HR is characterized by leaf lesions similar to those we observed in the necrotic F_1_ hybrids. To determine whether the ectopic immune response suggested by the gene expression profiles correlated with HR-like cell death, we stained leaves of hybrids and their parents with Trypan Blue, an indicator of dead cells. The degree and pattern of staining varied, but all necrotic F_1_ hybrids displayed greatly elevated levels of cell death compared with their parents at 16 °C (Figure 4B). These leaf lesioning phenotypes are similar to those observed in a class of mutants termed lesion mimics, which also suffer from autoimmune responses and ectopic cell death [28]. Some parent lines, Hh-0, Pla-1, Mrk-0, Uk-3, Uk-6, and Wei-1, showed occasional diffuse lesion-like staining, but this was mild, far less extensive than in the corresponding hybrids, and not associated with macroscopic symptoms (Figure 4B).

**Figure 5:**
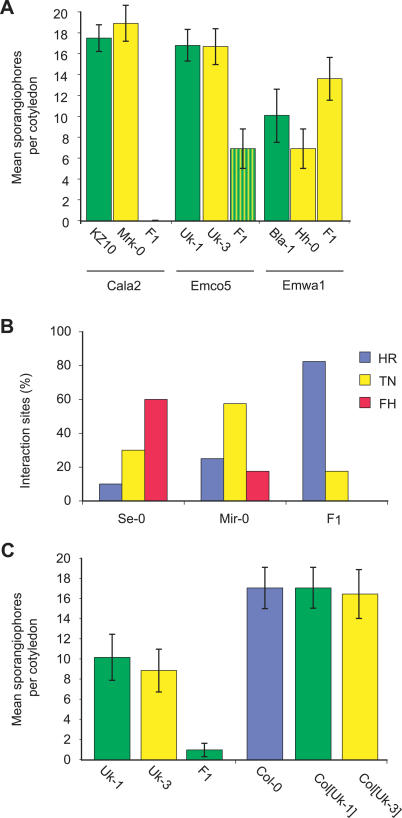
Susceptibility of Hybrids and Parental Accessions to H. parasitica Infection (A) Number of H. parasitica sporangiophores, scored 5 d (KZ10/Mrk-0, Uk-1/Uk-3, and Se-0/Hh-0) or 6 d (Bla-1/Hh-0) post-infection. H. parasitica strains used are indicated on the bottom. (B) Resistance and disease progression in Mir-0, Se-0, and their F_1_ hybrids. Since H. parasitica Noco2 sporulated only weakly on the parents, resistance was assessed by scoring interaction sites (Figure S2). HR sites indicate resistance, free hyphae sites (FH) indicate susceptibility, and trailing necrosis sites (TN) indicate an intermediate response. (C) H. parasitica Emco5 infection of Uk-1, Uk-3, their F_1_ hybrid, Col-0, and two Col-0 backcross lines. Col[Uk-1] carries the *DM2* region from Uk-1 on Chromosome 3, while Col[Uk-3] carries the *DM1* region from Uk-3 on Chromosome 5. Error bars in (A) and (C) indicate 95% confidence interval. All experiments were performed at least twice.

To confirm unambiguously that the microarray profiles reflect subthreshold activation of a functional immune response, we assayed the sensitivity of F_1_ hybrids and their respective parents to a common natural A. thaliana pathogen, the oomycete Hyaloperonospora parasitica [29]. To avoid complications due to the very different phenotypes of F_1_ hybrids and parents at 16 °C, we carried out infections at 20–23 °C. While the different F_1_ hybrids were asymptomatic with respect to ectopic cell death at this temperature (Figure S2), we knew that at least the Uk-1/Uk-3 hybrid already expressed a weakly activated molecular phenotype even at 23 °C (Figure 3A). For each combination tested, we first identified H. parasitica isolates to which both parents were susceptible. We then compared susceptibility of F_1_ hybrids and their parents to the different isolates. The Uk-1/Uk-3, KZ10/Mrk-0, and Mir-0/Se-0 F_1_ hybrids displayed enhanced disease resistance when compared with their respective parents (Figure 5A and 5B), indicating an increase in functional immune responses. In contrast, Bla-1/Hh-0 hybrids were at least as sensitive as their parents to the H. parasitica Emwa1 isolate (Figure 5A). The most likely explanation is that subthreshold ectopic immune responses are too weak at 20–23 °C in Bla-1/Hh-0 hybrids to increase resistance to H. parasitica infection.

Using the Uk-1/Uk-3 case as a model, we investigated whether the interaction between the two incompatible loci, or each locus individually, is associated with an ectopic, functional immune response. We tested lines in which the *DM1* and *DM2* regions from Uk-3 and Uk-1 were introgressed separately into Col-0, which yields normal offspring with both Uk-1 and Uk-3 (see above). The introgression lines on their own did not exhibit enhanced resistance to H. parasitica (Figure 5C); hence, the epistatic genetic interaction between *DM1* and *DM2,* which causes necrotic incompatibility at 16 °C, is also required for the increased disease resistance phenotype at 20–23 °C. We conclude that the hybrid necrosis cases we identified result from inappropriate activation of functional immune responses due to simple epistatic interactions.

To determine whether the observed immune system activation was caused by intrinsic genetic incompatibility, as opposed to hypersensitivity to microorganisms present in the environment, we grew several necrotic F_1_ hybrids in axenic conditions. We used severe class 3 hybrids (Uk-1/Uk-3, KZ10/Mrk-0, and REN11/Wei-1), since these develop symptoms sufficiently quickly to assay their phenotype in Petri dishes. Consistent with genetically programmed autoimmunity, these hybrids all displayed in sterile conditions necrosis symptoms that were similar to those seen when plants were grown on soil (not shown).

### Genetic Architecture of Hybrid Necrosis Cases in A. thaliana

A hallmark of hybrid necrosis in other plants is that it is normally genetically simple, like the Uk-1/Uk-3 example detailed above [2]. To determine whether this holds true for other cases in *A. thaliana,* we performed F_2_ segregation analyses of KZ10/Mrk-0 (also class 3), as well as two representatives of milder phenotypic classes, Bla-1/Hh-0 (class 2) and Mir-0/Se-0 (class 1). In each case, discrete phenotypic categories were observed in the F_2_ generation, with the original F_1_-like phenotype reappearing at high frequency, indicating a relatively simple genetic basis (Table 1). F_2_ populations also generally included plants with more severe phenotypes (growth arrest and lethality) at ratios suggesting that these individuals were homozygous for incompatible alleles.

Our mapping data for Uk-1/Uk-3 (see above) revealed that epistatic interaction between two unlinked semi-dominant loci, *DM1* and *DM2,* is sufficient to explain necrosis in this case. Double heterozygotes expressed hybrid necrosis, while homozygotes for the incompatible alleles suffered from growth arrest or lethality at 16 °C. When we grew F_2_ populations at 23 °C, we observed necrotic individuals at ratios consistent with these affected plants being partially or doubly homozygous at *DM1* and *DM2* (data not shown). This result implies that the temperature rescue of the phenotype is quantitative and can be overwhelmed by increased dosage of either incompatible allele. Similar results were observed for two other hybrids (Table 1). Thus, at higher temperatures, which suppress the F_1_ hybrid defects, the A. thaliana epistatic interactions cause milder recessive F_2_ “hybrid breakdown” problems rather than dominant F_1_ incompatibilities. These results underscore the importance of environmental milieu for the expression of hybrid necrosis.

## Discussion

Our results demonstrate that aberrant activation of the plant immune system, due to epistatic interactions between alleles that are harmless in their native genetic context, can have highly deleterious consequences for F_1_ hybrid progeny, even among strains within a species. For one case, we demonstrate that this is caused by *DM1,* an allele of an *NB-LRR* gene.

F_1_ incompatibilities that are phenotypically similar to the cases of A. thaliana hybrid necrosis we describe have been observed in progeny of many intra- and interspecific crosses [2,4,23,30–33], suggesting that a common molecular mechanism may underlie hybrid necrosis across diverse species. In support of this hypothesis, interspecific hybrid necrosis in tobacco has been linked to activation of genes associated with pathogen responses [8]. Additional support for a connection between hybrid necrosis and mechanisms of disease resistance comes from the analysis of the tomato gene *Cf-2*. The *Cf-2* locus, which was originally identified in Solanum pimpinellifolium as conferring resistance against *Cladosporum fulvum,* can cause autonecrosis (a milder syndrome broadly similar to hybrid necrosis) when introgressed into domesticated tomato, S. lycopersicum [34]. However, autonecrosis occurs only in plants homozygous for S. lycopersicum alleles at a second gene, *RCR3.* Based on this, and the observation that *R* gene alleles can cause autonecrosis when transiently expressed in tobacco, Wulff, Jones, and colleagues presciently suggested that constitutive *R* gene activation in hybrids might contribute to the maintenance of interspecific postzygotic hybridization barriers [35].

*Cf-2*-triggered autonecrosis is not accompanied by severe morphological defects and is thus less deleterious than the class 2 and 3 A. thaliana cases. Nevertheless, the fundamental parallels in phenotype and genetic architecture imply that these differences are likely a matter of degree. We therefore propose that the severe A. thaliana hybrid necrosis cases represent the phenotypic extremes of a quantitative distribution of epistatic interactions that often involve *R* gene homologs. Notably, the *Cf-2* gene encodes a type of R protein that does not belong to the NB-LRR class, indicating that hybrid necrosis and related phenomena may involve *R* genes more generally, and that the causes are not restricted to any particular class of disease resistance genes.

Known functional and evolutionary properties of the plant immune system [11,36] provide a clear mechanistic framework within which to explain the widespread occurrences of hybrid necrosis observed throughout flowering plants and to understand how simple epistatic interactions causing hybrid necrosis could arise repeatedly within a species. *NB-LRR* genes are known to be highly diverse, and this has been interpreted as indicative of positive selection [13,37–39]. Furthermore, proper regulation of NB-LRR protein signaling depends on combinations of NB-LRR proteins and additional host proteins, with which they genetically, and likely physically, interact [11,36]. Hence, both NB-LRR-mediated disease resistance and ectopic NB-LRR activation can result in cell death symptoms similar to those associated with hybrid necrosis.

We have demonstrated that an *NB-LRR* gene in the Uk-3 accession (the *DM1* locus) is sufficient to cause genetic incompatibility with the *DM2* locus from the Uk-1 accession. Though we do not yet know which gene or gene product from Uk-1 interacts with *DM1,* it is intriguing that, at least in the Col-0 reference genome, the *DM2* interval also contains two annotated *NB-LRR* genes. No other gene known to be involved in pathogen response is found in the *DM2* interval. This raises the tantalizing possibility that aberrant interactions between diverged R proteins encoded by *NB-LRR* genes could cause hybrid necrosis when brought into the same genomic context by hybridization. Thus, co-evolution among *R* gene products could be important to prevent ectopic activation. Consistent with an interaction among *R* genes, there are several cases in which two *R* genes are required for disease resistance [40–42]. The mechanistic basis, based on precedence in animals [43] and evidence in plants [44], could lie in heterodimeric interactions of NB-LRR proteins during pathogen recognition [45]. However, because the causal gene in the *DM2* interval has not yet been identified, an alternative possibility is that the *DM1* NB-LRR protein normally associates with a cellular partner encoded by the *DM2* locus whose homeostasis it monitors, consistent with the “guard hypothesis” for NB-LRR function [11]. The maintenance of such interactions would also require co-evolution of the two partners, with a failure of guard–guardee interaction resulting in dose-dependent hybrid necrosis as we observe here.

Deleterious epistatic interactions involving diverse plant disease resistance genes and variable haplotypes at a second host locus could thus arise as by-products of selection related to host–pathogen conflicts. Indeed, there is a striking association in many plant taxa between selection for increased disease resistance and the occurrence of hybrid necrosis [2].

The fact that hybrid necrosis is associated with numerous interspecific incompatibilities [2,4,23,30–33] suggests that causal alleles could, at least occasionally, become fixed between populations. Indeed, the genetic architecture of the A. thaliana cases bears a striking similarity to that of Dobzhansky-Muller incompatibilities, as do many other examples of intra- and interspecies plant hybrid necrosis [2]. Though *R* genes are thought to be often under balancing selection, and consequently unlikely to go to fixation, there is growing evidence that *R* gene evolution is more complex, and that many *R* genes exist in other evolutionary states, including regional selective sweeps [39] and rapid allelic expansion consistent with arms races [37]. Further examples of hybrid necrosis where causal alleles occur at different levels of fixation need to be characterized to determine whether *R* genes are also involved in these cases, and whether bona fide gene flow barriers might arise from *R*-gene-mediated hybrid necrosis.

In conclusion, A. thaliana presents a useful model to understand a widespread genetic incompatibility syndrome, hybrid necrosis. The mechanisms underlying hybrid necrosis are potentially diverse, despite the apparently common involvement of the immune system. We argue, however, that a model encompassing several different incompatibilities will prove immensely valuable. We have already shown that at least one hybrid necrosis case in A. thaliana is caused by a member of the *NB-LRR* family, which constitutes the most common type of *R* genes in plants. Together with previous findings, this raises many important questions. How often is hybrid necrosis caused by *R* gene homologs? Do most cases involve *NB-LRR* genes? What are the biochemical mechanisms whereby *R* genes cause deleterious interactions with other genotypes? And finally, if hybrid necrosis contributes to gene flow barriers, how important is it compared to other phenomena such as prezygotic isolation or hybrid sterility?

## Materials and Methods

### Plant material and growth conditions.

Seeds for A. thaliana accessions were obtained from the European Arabidopsis Stock Centre (http://www.arabidopsis.info/) and the Arabidopsis Biological Resource Center (http://www.biosci.ohio-state.edu/~plantbio/Facilities/abrc/abrchome.htm). Stock numbers of accessions used are given in Table S3. Uk-6 through Uk-14 were collected on 28 May 2005 in the town of Umkirch, Germany. All seeds were stratified in the dark in 0.1% agarose for 4–7 d at 4 °C prior to planting on soil. Late-flowering accessions were induced to germinate for 1 d at 23 °C and then vernalized for 6 wk as seedlings in 4 °C short-day conditions (8 h light) under about 50 μmol m^−2^ s^−1^ light fluence. All plants were grown in long-day conditions (16 h light) in controlled temperature growth chambers with a variability of about ±0.1 °C at 16 °C or 23 °C, with 65% relative humidity. Chambers were illuminated with a 1:1 mix of Cool White and Gro-lux wide-spectrum fluorescent lights with a fluence rate of 125–175 μmol m^−2^ s^−1^.

### Uk-1/Uk-3 backcross lines.

Backcross lines were generated for the Uk-1 and Uk-3 incompatibility regions by crossing Uk-1 × Uk-3 F_1_ plants with Col-0. Sick plants were selected at 16 °C for further backcrossing in each generation. After three generations of backcrossing, the lines were genotyped using three markers per chromosome, with additional markers in the incompatible regions. Two lines were selected for further analysis: Col[Uk-1] carries a region from Uk-1 on Chromosome 3 and also retains heterozygous regions on the bottom of Chromosome 1 and in the middle of Chromosome 2, and Col[Uk-3] carries the incompatibility locus from Uk-3 on Chromosome 5 and retains a heterozygous region at the bottom of Chromosome 3 (this does not extend to the Uk-1 incompatibility region).

### Haplotype analysis of *DM1* region.

Fragments linked to *DM1* were generated with the following primer sets: 5′ TGC TGT TAA TTT GCG AGT GC 3′ and 5′ AAG ATA CGT TGC CAC CAA GG 3′ (*AT5G41760;* produces a 1,295-bp fragment identical in Nc-1, Uk-3, and Uk-6), and 5′ GCA GTG CAG GTT CAC TTC A 3′ and 5′ ACA GGT GCT GAA GCA TAC GAA 3′ (*AT5G41700;* produces a 980-bp fragment identical in Nc-1, Uk-3, and Uk-6). Sequences within *DM1* are highly similar between the accessions.

### Microarray analysis.

Plants used for microarray analysis were grown on soil under long-day conditions. Uk-1/Uk-3 parents and F_1_ hybrids were grown at 23 °C short days (8 h light, 16 h dark) for 27 d, then plants were shifted to 16 °C short days for 5 d. Tissue for RNA was harvested at day 0 and day 5. Only the central part of each plant, including the meristem and youngest leaves, was used. KZ10/Mrk-0 parents and F_1_ hybrids were grown at 23 °C long days for 12 d then shifted to 16 °C and harvested after 2 d. Bla-1/Hh-0 and Mir-0/Se-0 parents and F_1_ hybrids were grown at 16 °C for 4 wk. The entire aerial portions of the plants were used for KZ10/Mrk-0, Bla-1/Hh-0, and Mir-0/Se-0.

RNA samples were processed in triplicate from pooled samples of eight to ten plants per genotype where whole plants were used, and 40 plants when only apices were used (Uk-1/Uk-3). Total RNA was prepared using the RNeasy Plant RNA extraction kit (Qiagen, http://www.qiagen.com/). Probes for microarrays were made and labeled using the Message Amp II Biotin kit (Ambion, http://www.ambion.com/). Hybridization, washing, and scanning were done following Affymetrix (http://www.affymetrix.com/) guidelines using a Hybridization Oven 640, a Fluidics Station 450, and a GeneChip Scanner 3000.

Array data were analyzed using LogitT [46] and Rank Product [47] at a *p*-value of 0.05. Genes that were significantly different in any given pairwise comparison under both tests were used in further analysis. Data were normalized across arrays using gcRMA analysis in GeneSpring 5.1 (Silicon Genetics, now Agilent Technologies, http://www.chem.agilent.com/). PCA was also performed in GeneSpring. Gene Ontology (http://www.geneontology.org/) was examined for the Uk-1/Uk-3 hybrid data using GOstat (http://gostat.wehi.edu.au/).

### Histology.

Trypan Blue staining was performed as previously described [29].

### Pathology.

To assay H. parasitica sporangiophore growth, 8- to 10-d-old seedlings were inoculated with 5 × 10^4^ spores/milliliter of H. parasitica isolates Cala2, Emco5, Noco2, and Emwa1 (Table S6). Sporangiophores were counted at 4–5 dpi as described [48]. For the cell death assay, 8-d-old Mir-0, Se-0, and hybrid seedlings were inoculated with 5 × 10^4^ spores/milliliter of the rare sporulator H. parasitica Noco2. Forty infected cotyledons per genotype were stained with Trypan Blue at 7 dpi, and interaction sites were assayed as described [49].

### Mapping and SNP genotyping.

For mapping, SNPs were selected from a large available dataset of sequence reads from 96 A. thaliana accessions [50]. One set of 289 SNPs chosen maximizes polymorphism of random accessions with Col-0. These SNP markers were used to generate MassARRAY markers and were tested on Uk-1 and Uk-3 at Genaissance Pharmaceuticals (now Cogenics, http://www.cogenics.com/). The MassARRAY system uses MALDI-TOF mass spectrometry to determine SNPs and other small-scale polymorphisms after single-primer extension on PCR-amplified DNA fragments. Of the 289 SNPs, 64 were informative for the Uk-1 × Uk-3 combination and were used to genotype 91 F_2_ and backcross individuals (70 F_2_ and 21 backcross lines) showing the F_1_-like phenotype at 16 °C, and 90 plants showing a normal phenotype (69 F_2_ and 21 backcross lines). MassARRAY SNP markers as described above were also used to identify differences among strains. A total of 311 markers that show intermediate frequency in a worldwide sample were selected and tested on Uk-1 to Uk-14, Nc-1, and 81 other randomly selected strains, and genotyped by Sequenom (http://www.sequenom.com/).

Markers used for fine mapping were generated by PCR amplification and dideoxy sequencing of 0.5- to 1.5-kb-long fragments from Uk-1 and Uk-3. Primers were designed from the Col-0 reference sequence using Primer3 (http://frodo.wi.mit.edu/cgi-bin/primer3/primer3_www.cgi). Sequence differences between Uk-1 and Uk-3 were used to generate PCR-based length polymorphism or restriction polymorphism markers. These were used to identify recombinants among 1,000 F_2_ plants showing the F_1_-like phenotype at 16 °C.

QTL mapping and testing for epistatic interaction was performed using the R/qtl package [51]. This freely available software package is implemented as an add-on package for the command-line-based, open-source statistical software R (http://www.R-project.org/). For QTL mapping, phenotypes were entered as 1 (no phenotype) and 2 (necrotic phenotype). Lod scores were calculated with a single-QTL model using the function “scanone.” The lod score significance threshold was established using 1,000 permutations. The epistatic interactions were calculated with the function “scantwo.” In both functions the standard expectation-maximization algorithm was used for “method.”

### Fosmid libraries.

Fosmid libraries were prepared using 20-μg Uk-1 and Uk-3 genomic DNA prepared using the Masterpure Plant Leaf DNA Purification Kit (Epicentre Biotechnologies, http://www.epibio.com/). Libraries were produced using the Copy Control Fosmid Library Production Kit (Epicentre Biotechnologies) following the manufacturer's protocol. Fosmid libraries were replica-plated on Hybond-N+ nitrocellulose membranes (Amersham, http://www.amersham.com/). Colonies were lyzed, denatured, and fixed directly to the filters and cross-linked in a UV-Stratalinker 2400 (Stratagene, http://www.stratagene.com/) with default settings. Digoxigenin-labeled probes were generated by PCR using the PCR DIG Probe Synthesis Kit (Roche, http://www.roche.com/). Filters were hybridized and screened using standard hybridization procedures. Two digoxigenin-labeled probes were used, one on each end of the interval, in *AT5G41700* (primers: 5′ GCA GTG CAG GTT CAC TTC AA 3′ and 5′ CAG GTG CTG AAG CAT ACG AA 3′) and in *AT5G41760* (5′ TGC TGT TAA TTT GCG AGT GC 3′ and 5′ AAG ATA CGT TGC CAC CAA GG 3′). Detection was performed using anti-digoxigenin antibody (Roche) and CSPD chemiluminescent substrate (Applied Biosystems, http://www.appliedbiosystems.com/) following manufacturer protocols. Membranes were exposed to X-ray film (Kodak, http://www.kodak.com/) for 15 min to overnight as needed. Positive clones were streaked from original plates on fresh LB plates (12.5 μg/ml chloramphenicol) and assayed by PCR for confirmation.

Positive clones were grown in liquid 2YT medium containing 12.5 μg/ml chloramphenicol and induced to high copy number using 1:100 CopyControl Induction solution (Epicentre Biotechnologies). Fosmid DNA was prepared from 200 ml of induced culture using a large construct preparation kit (Qiagen). Fosmid DNA (5–10 μg) was randomly sheared to less than 10 kb using nebulizers (Invitrogen, http://www.invitrogen.com/) and cloned into pCR4Blunt TOPO vector (Invitrogen). Sub-clone colonies were blue-white selected, and plasmid DNA was isolated from 768 positive clones using the MagAttract 96 Miniprep Core Kit (Qiagen) on a Biorobot 8000 (Qiagen). Clones were end-sequenced using T3 and T7 primers. Sequence reads were assembled into contigs using Phred Phrap Consed software (http://www.phrap.org/phredphrapconsed.html).

### AmiRNA rescue and genomic complementation.

Constructs for constitutive expression of amiRNAs from the CaMV 35S promoter were designed and prepared as previously described [15]. We generated amiRNA constructs targeting *AT5G41700, AT5G41720,* and *AT5G41730,* and three different constructs targeting *AT5G41740/AT5G41750,* always using sequences known to be present in Uk-3. The genomic complementation construct was generated by cloning an 8-kb HincII/XbaI fragment from a Uk-3 fosmid clone into the pGreen IIS vector [52].

All constructs were transformed into Uk-3, Col-0, and/or Uk-1 using Agrobacterium tumefasciens (strain ASE) by dip transformation. T_1_ plants were selected on half-strength MS plates containing 100 μg/ml kanamycin. T_1_ plants were transplanted onto soil and grown at 23 °C. amiRNA-expressing T_1_ plants were crossed with Uk-1, and progeny from these crosses were grown at 16 °C and scored for rescue phenotypes. A randomly selected rescuing line (KB142–2 [Uk-3] × Uk-1) was used for microarray analysis as described above. Whole aerial portions of 28-d-old sick and healthy plants were used, grouped in three pools of ten plants per phenotype.

## Supporting Information

Figure S1 Alignment of *DM1* from Uk-3 with Col-0 Alleles (3.0 MB AI).

Figure S2 Trypan Blue Staining of Seedlings (5.0 MB AI).

Figure S3 Expression of Selected Pathogen Response Markers in Hybrids (1.2 MB AI).

Table S1 Significantly Overrepresented Gene Ontology Terms (55 KB DOC)

Table S2 Gene Categories in Differentially Expressed Genes (103 KB DOC)

Table S3 Accession Numbers of Lines (319 KB DOC)

Table S4 Crosses Performed and Hybrid Phenotypes (1.0 MB DOC)

Table S5 Intercrossability of Parents of Several Incompatibilities (46 KB DOC)

Table S6 H. parasitica Resistance of Hybrids and Parents (50 KB DOC)

### Accession Numbers

The Arabidopsis Biological Resource Center and European Arabidopsis Stock Centre accession numbers for the lines discussed in this paper are given in Table S3. The ArrayExpress (http://www.ebi.ac.uk/arrayexpress/) accession numbers for the new microarray data discussed in this paper are E-ATMX-24, E-ATMX-25, E-ATMX-26, and E-ATMX-27. The GenBank (http://www.ncbi.nlm.nih.gov/Genbank/) accession numbers for the fosmid sequences are EU012335, EU012336, and EU012337.

## Abbreviations

amiRNA: artificial microRNA
HR: hypersensitive response
PCA: principal component analysis
QTL: quantitative trait locus
SNP: single nucleotide polymorphism
