# Supplementary material for: Autoimmune Response as a Mechanism for a Dobzhansky-Muller-Type Incompatibility Syndrome in Plants
Source: PLoS Biol. 2007 Sep 4;5(9):e236. doi: 10.1371/journal.pbio.0050236 (PMC1964774; doi:10.1371/journal.pbio.0050236)
Supplement: Supplementary file 1 [file pbio.0050236.sg001.pdf]

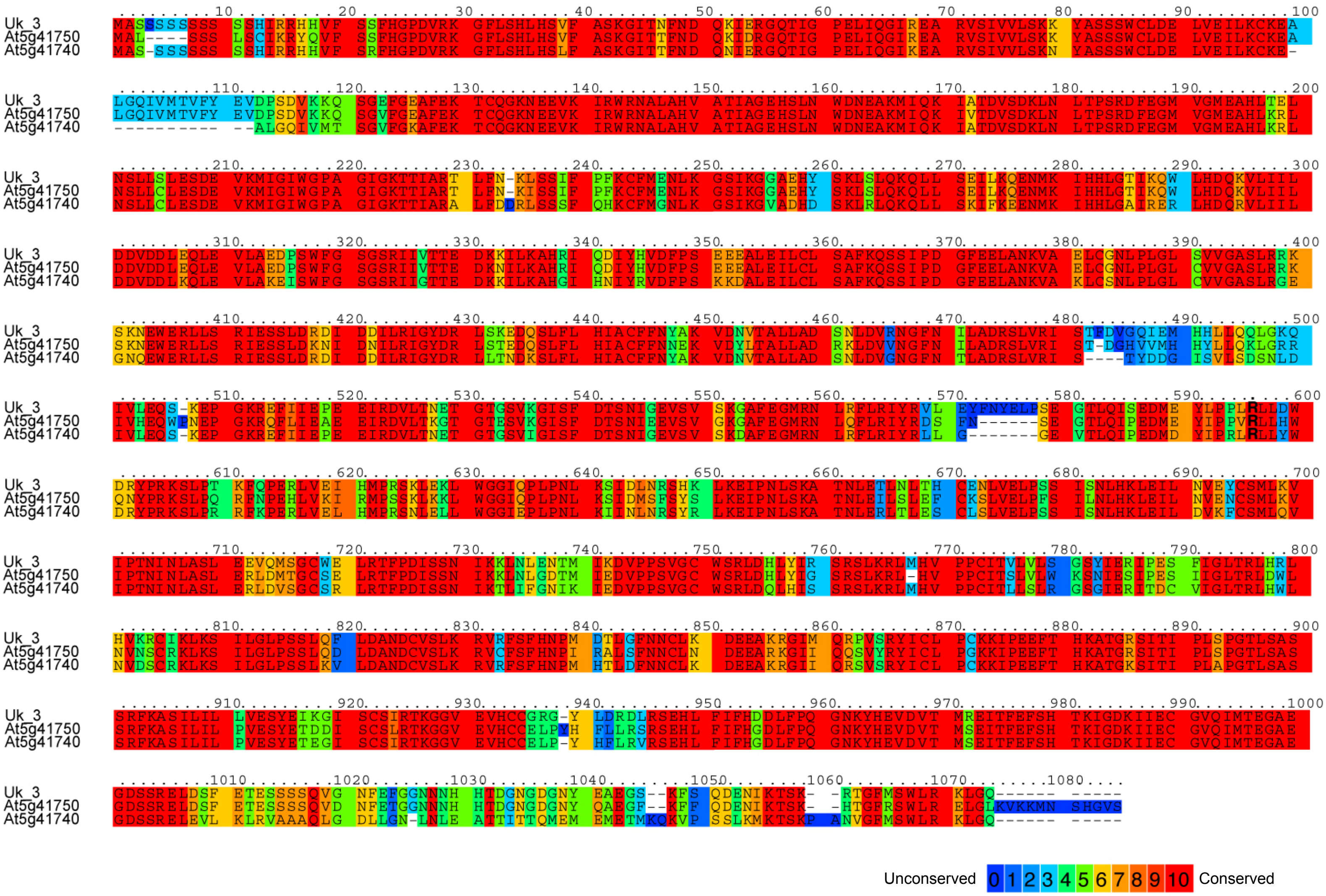
Uk_3
At5g41750
At5g41740
Unconserved
0 1 2 3 4 5 6 7 8 9 10
Conserved
