# Supplementary material for: Autoimmune Response as a Mechanism for a Dobzhansky-Muller-Type Incompatibility Syndrome in Plants
Source: PLoS Biol. 2007 Sep 4;5(9):e236. doi: 10.1371/journal.pbio.0050236 (PMC1964774; doi:10.1371/journal.pbio.0050236)
Supplement: Supplementary file 2 [file pbio.0050236.sg002.pdf]

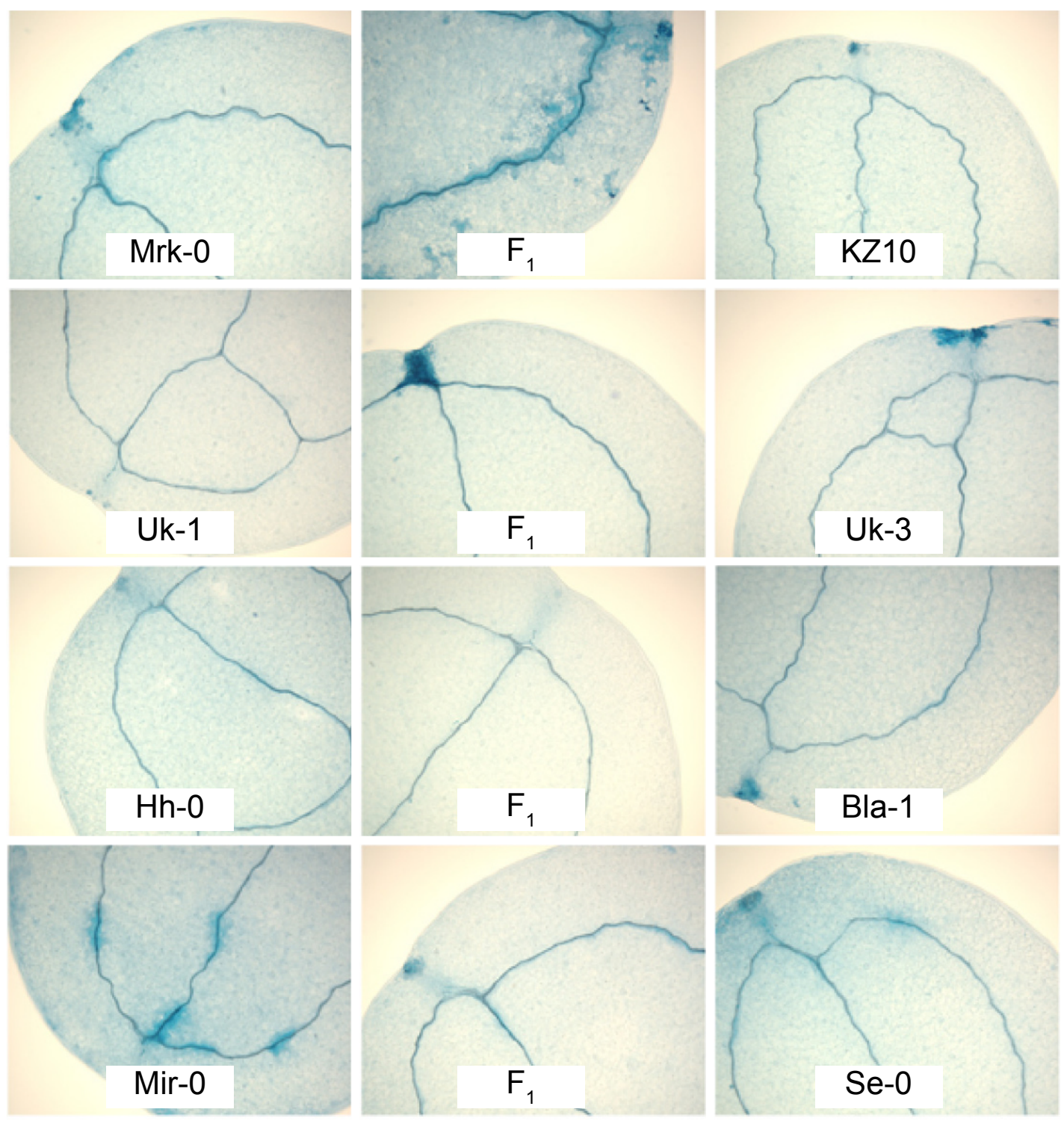
Mrk-0
F_1
KZ10
Uk-1
F_1
Uk-3
Hh-0
F_1
Bla-1
Mir-0
F_1
Se-0
