# Supplementary material for: Autoimmune Response as a Mechanism for a Dobzhansky-Muller-Type Incompatibility Syndrome in Plants
Source: PLoS Biol. 2007 Sep 4;5(9):e236. doi: 10.1371/journal.pbio.0050236 (PMC1964774; doi:10.1371/journal.pbio.0050236)
Supplement: Supplementary file 3 [file pbio.0050236.sg003.pdf]

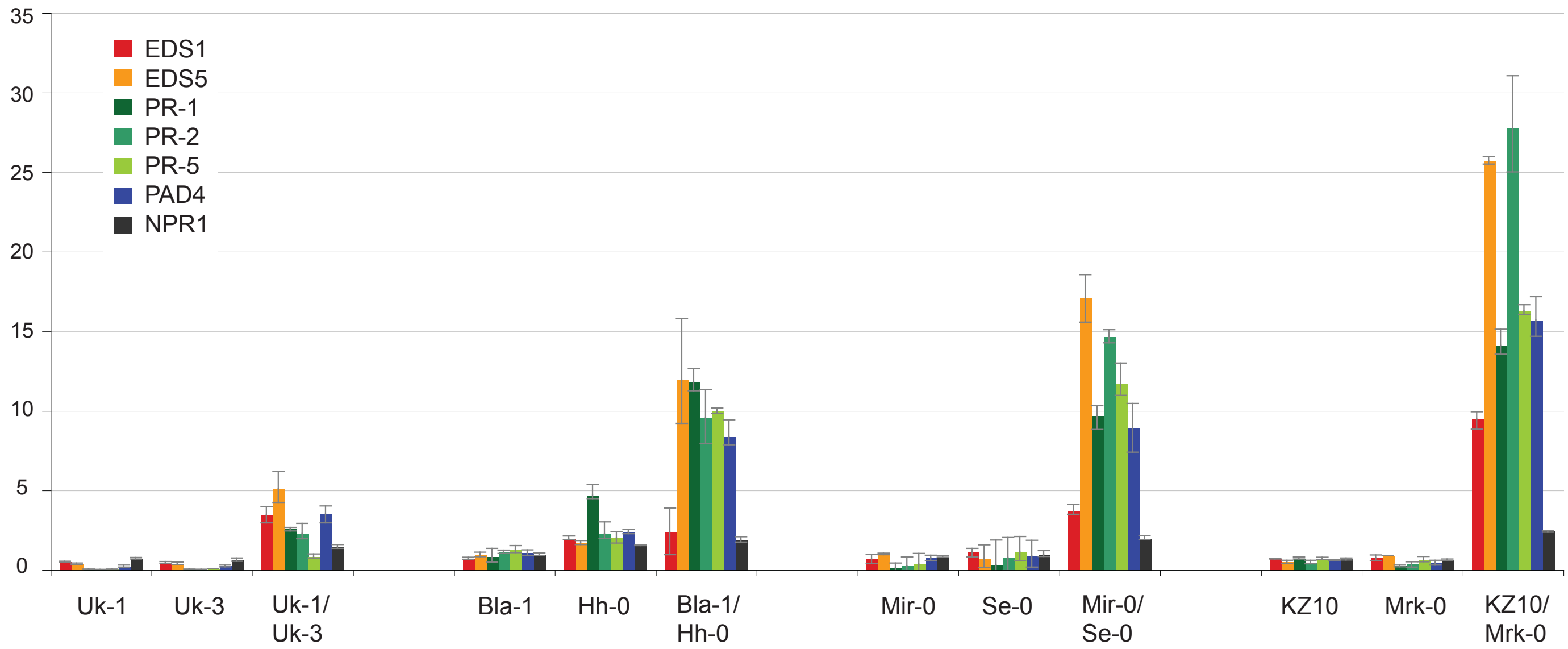

35
30
25
20
15
10
5
0
EDS1
EDS5
PR-1
PR-2
PR-5
PAD4
NPR1
Uk-1
Uk-3
Uk-1/ Uk-3
Bla-1
Hh-0
Bla-1/ Hh-0
Mir-0
Se-0
Mir-0/ Se-0
KZ10
Mrk-0
KZ10/ Mrk-0
