# Supplementary material for: Autoimmune Response as a Mechanism for a Dobzhansky-Muller-Type Incompatibility Syndrome in Plants
Source: PLoS Biol. 2007 Sep 4;5(9):e236. doi: 10.1371/journal.pbio.0050236 (PMC1964774; doi:10.1371/journal.pbio.0050236)
Supplement: Supplementary file 4 [file pbio.0050236.st001.doc]

**Table S1** Significantly over-represented GO terms (among genes different in Uk-1/Uk-3 F_1_ versus parents, but not between parents).

| **GO process** | **# genes Uk F_1_ list** | **# in *At* genome** | **P-value** |
| --- | --- | --- | --- |
| Systemic acquired resistance (SAR) | 3 | 5 | 0.002 |
| response to biotic stimulus | 15 | 411 | 0.002 |
| response to pathogen | 8 | 131 | 0.003 |
| response to other organism | 9 | 182 | 0.004 |
| response to pest, pathogen or parasite | 8 | 147 | 0.005 |
| response to stimulus | 21 | 953 | 0.007 |
| defense response | 11 | 327 | 0.013 |
| anthocyanin biosynthesis | 2 | 5 | 0.016 |
| protein targeting to vacuole | 2 | 5 | 0.016 |
| flavonoid biosynthesis | 3 | 20 | 0.016 |
| flavonoid metabolism | 3 | 20 | 0.016 |
| response to stress | 12 | 420 | 0.016 |
| defense response, response to pathogen, incompatible interaction | 5 | 78 | 0.016 |
| defense response to pathogen | 5 | 85 | 0.022 |

Notes: Statistically significant over-representation of gene ontology (GO) term categories was calculated in GoStat ([http://gostat.wehi.edu.au](http://gostat.wehi.edu.au/)). P-values are Fisher’s Exact test p-values corrected for multiple testing with Benjamini correction as implemented in GoStat. GO term annotation was based on the latest TAIR release ([http://www.arabidopsis.org](http://www.arabidopsis.org/)).
