# Supplementary material for: Autoimmune Response as a Mechanism for a Dobzhansky-Muller-Type Incompatibility Syndrome in Plants
Source: PLoS Biol. 2007 Sep 4;5(9):e236. doi: 10.1371/journal.pbio.0050236 (PMC1964774; doi:10.1371/journal.pbio.0050236)
Supplement: Supplementary file 5 [file pbio.0050236.st002.doc]

**Table S2** Representation of gene categories in list of genes differentially regulated in at least one hybrid versus its parents.

| **Category** | **on array** | **in list** | **expected** | **fold-**  **excess** |
| --- | --- | --- | --- | --- |
| **Total** | **22747** | **1080** |  |  |
| chitinase | 8 | 6 | 0.4 | 15.8 |
| BON1/copine | 10 | 4 | 0.5 | 8.4 |
| trypsin inhibitor | 10 | 3 | 0.5 | 6.3 |
| tropinone reductase | 14 | 4 | 0.7 | 6.0 |
| harpin-induced family | 15 | 4 | 0.7 | 5.6 |
| glutamate receptor | 19 | 5 | 0.9 | 5.5 |
| WRKY transcription factor | 62 | 16 | 2.9 | 5.4 |
| glutathione S-transferase, putative | 46 | 11 | 2.2 | 5.0 |
| receptor-like kinase | 20 | 4 | 0.9 | 4.2 |
| MutT/nudix | 21 | 4 | 1.0 | 4.0 |
| heavy-metal associated | 39 | 7 | 1.9 | 3.8 |
| FAD binding domain | 38 | 6 | 1.8 | 3.3 |
| xyloglucan | 32 | 5 | 1.5 | 3.3 |
| flavin-containing monooxygenase family protein / FMO family protein | 26 | 4 | 1.2 | 3.2 |
| peroxidase | 65 | 10 | 3.1 | 3.2 |
| NAM | 87 | 13 | 4.1 | 3.1 |
| Oxidoreductase, 2OG-Fe(II) oxygenase family protein | 60 | 8 | 2.8 | 2.8 |
| pathogenesis-related | 30 | 4 | 1.4 | 2.8 |
| calcium/calmodulin | 203 | 27 | 9.6 | 2.8 |
| lipase | 39 | 4 | 1.9 | 2.2 |
| cytochrome P450 | 213 | 21 | 10.1 | 2.1 |
| glycosyl hydrolase | 176 | 17 | 8.4 | 2.0 |
| S-locus protein kinase | 32 | 3 | 1.5 | 2.0 |
| disease resistance family | 206 | 19 | 9.8 | 1.9 |
| plant defensin | 11 | 1 | 0.5 | 1.9 |
| leucine-rich repeat | 295 | 23 | 14.0 | 1.6 |
| GDSL-motif lipase/hydrolase family protein | 77 | 6 | 3.7 | 1.6 |
| protein kinase | 467 | 35 | 22.2 | 1.6 |
| nodulin | 69 | 5 | 3.3 | 1.5 |
| heat shock | 57 | 4 | 2.7 | 1.5 |
| Senescence-associated (SAG) | 43 | 3 | 2.0 | 1.5 |
| glycoside hydrolase | 46 | 3 | 2.2 | 1.4 |
| LEA | 36 | 2 | 1.7 | 1.2 |
| glycine-rich protein | 90 | 4 | 4.3 | 0.9 |
| expressed protein | 4256 | 172 | 202.1 | 0.9 |
| AP2 domain | 78 | 3 | 3.7 | 0.8 |
| DEAD-box | 54 | 2 | 2.6 | 0.8 |
| zinc finger protein | 547 | 20 | 26.0 | 0.8 |
| bHLH | 83 | 2 | 3.9 | 0.5 |
| glycosyl transferase | 104 | 2 | 4.9 | 0.4 |
| DnaJ | 96 | 1 | 4.6 | 0.2 |
| transducin-related | 109 | 1 | 5.2 | 0.2 |
| F-box | 321 | 2 | 15.2 | 0.1 |
| ethylene-responsive | 36 | 0 | 1.7 | 0.0 |
| MADS | 65 | 0 | 3.1 | 0.0 |
| B3 family transcription factor | 41 | 0 | 1.9 | 0.0 |

Note: The gene list consists of 1,080 genes (4.7% of genes on the microarray), which are differentially expressed in at least one of the four hybrids tested with respect to its parents.
