# Supplementary material for: Autoimmune Response as a Mechanism for a Dobzhansky-Muller-Type Incompatibility Syndrome in Plants
Source: PLoS Biol. 2007 Sep 4;5(9):e236. doi: 10.1371/journal.pbio.0050236 (PMC1964774; doi:10.1371/journal.pbio.0050236)
Supplement: Supplementary file 6 [file pbio.0050236.st003.doc]

**Table S3** Accession numbers of lines used.

| Name | NASC | ABRC |
| --- | --- | --- |
| Aa-0 | N935 |  |
| Ak-1 | N939 |  |
| ANH1 | N6607 |  |
| Ba-1 | N952 |  |
| Bay-0 |  | CS22676 |
| Bch-3 | N959 |  |
| Bch-4 | N961 |  |
| Bd-0 | N963 |  |
| Be-1 | N967 |  |
| BG1 | N22341 | |
| BG2 | N22342 |  |
| BG5 | N22345 |  |
| BG7 | N22347 |  |
| BG9 | N22349 |  |
| Bl-1 | N969 |  |
| Bla-1 | N971 |  |
| Bla-11 | N985 |  |
| Bla-12 | N987 |  |
| Bla-14 | N989 |  |
| Bla-2 | N973 |  |
| Bla-3 | N975 |  |
| Bla-4 | N977 |  |
| Bla-5 | N978 |  |
| Bla-6 | N980 |  |
| Blh-1 | N6645 |  |
| Blh-2 | N6657 |  |
| Bor-4 |  | CS22677 |
| Br-0 | N995 |  |
| Bs-1 | N997 |  |
| Bs-2 | N6628 |  |
| Bs-5 | N1001 |  |
| Bsch-0 | N1003 |  |
| Bsch-1 |  |  |
| Bsch-2 | N1005 |  |
| Bu-0 | N1007 |  |
| Bu-11 | N1025 |  |
| Bu-13 | N1027 |  |
| Bur-0 | N1029 |  |
| C24 |  | CS22680 |
| Ca-0 | N1061 |  |
| Cal-0 | N1063 |  |
| Can-0 | N1065 |  |
| Cen-0 | N1067 |  |
| Cha-0 | N1069 |  |
| Chi-0 | N1073 |  |
| Chi-1 | N1075 |  |
| Chi-2 | N1077 |  |
| CIBC1 | N22220 |  |
| CIBC10 | N22229 |  |
| Cit-0 | N1081 |  |
| Cnt-1 | N6921 |  |
| Co-1 | N1085 |  |
| Co-3 | N1089 |  |
| Co-4 | N1091 |  |
| Col-0 | N1093 |  |
| CSHL1 | N22419 |  |
| Ct-1 | N1095 |  |
| Cvi-0 |  | CS22682 |
| Db-0 | N1101 |  |
| Db-1 | N1103 |  |
| Db-2 | N6679 |  |
| Di-2 | N1111 |  |
| Dr-0 | N1115 |  |
| Dra-0 | N1117 |  |
| Dra-1 | N1119 |  |
| Edi-0 | N1123 |  |
| Ei-2 | N1125 |  |
| Ei-4 | N1127 |  |
| Ei-5 | N6691 |  |
| Ei-6 | N1131 |  |
| Eil-0 | N1133 |  |
| El-0 | N1135 |  |
| Ema-1 | N1681 |  |
| En-1 | N1137 |  |
| En-2 | N1139 |  |
| Enk-T | N6176 |  |
| Ep-0 | N1141 |  |
| Er-0 | N1143 |  |
| Est-0 | N1149 |  |
| Est-1 | N1151 |  |
| Estland | N6173 |  |
| Et-0 | N1153 |  |
| Fe-1 | N1155 |  |
| Fei-0 |  | CS22684 |
| Fi-0 | N1157 |  |
| Fi-1 | N1159 |  |
| FM11 | N22392 |  |
| Fr-2 | N1169 |  |
| Fr-3 | N1171 |  |
| Fr-4 | N1173 |  |
| Fr-5 | N1175 |  |
| Fr-6 | N1177 |  |
| Fr-7 | N1179 |  |
| Ga-0 | N1181 |  |
| Ga-2 | N1183 |  |
| Gd-1 | N1185 |  |
| Ge-1 | N1189 |  |
| Ge-2 | N1191 |  |
| Gie-0 | N1193 |  |
| Gö-0 | N1195 |  |
| Gö-2 | N1197 |  |
| GOT1 | N22277 |  |
| GOT10 | N22286 |  |
| GOT7 |  | CS22685 |
| Gr-1 | N1199 |  |
| Gr-2 | N1201 |  |
| Gr-3 | N1203 |  |
| Gr-5 | N1207 |  |
| Gr-6 | N6728 |  |
| Gre-0 | N1211 |  |
| Gü-0 | N1213 |  |
| Gü-1 | N1215 |  |
| Gy-0 | N1217 |  |
| Ha-0 | N1219 |  |
| Hau-0 | N1221 |  |
| Hh-0 | N1225 |  |
| Hi-0 | N1227 |  |
| Hl-0 | N1229 |  |
| Hl-2 | N1231 |  |
| Hl-3 | N1233 |  |
| Hn-0 | N1235 |  |
| Hodja-Obi | N6178 |  |
| HR14 | N22213 |  |
| HR15 | N22214 |  |
| HR5 | N22205 |  |
| HR8 | N22208 |  |
| Hs-0 | N1237 |  |
| HS1 | N22351 |  |
| In-0 | N1239 |  |
| Is-0 | N1241 |  |
| Is-1 | N1243 |  |
| Je-0 | N1247 |  |
| Jl-1 | N1249 |  |
| Jl-2 | N1251 |  |
| Jm-1 | N1261 |  |
| Js-0 | N1671 |  |
| Kb-0 | N1269 |  |
| Kil-0 | N1271 |  |
| Kin-0 | N1273 |  |
| Kl-0 | N1275 |  |
| Kl-1 | N1277 |  |
| Kn-0 | N1287 |  |
| KNO1 | N22401 |  |
| Kondara | N6175 |  |
| Kro-0 | N1301 |  |
| KZ10 | N22442 |  |
| KZ11 | N22443 |  |
| La-1 | N1303 |  |
| Lan-0 | N1305 |  |
| Le-0 | N1309 |  |
| Ler-1 | N6928 |  |
| 4nLer | N3900 |  |
| Li-10 | N6911 |  |
| Li-2 | N6908 |  |
| Li2:1 | N1315 |  |
| Lip-0 | N1337 |  |
| Ll-0 | N1339 |  |
| Ll-11 | N1651 |  |
| Ll-2 | N1343 |  |
| Lm-2 | N1345 |  |
| Lö-1 | N1347 |  |
| Lö-2 | N1349 |  |
| Lov-5 |  | CS22695 |
| M7943S | N6186 |  |
| Ma-0 | N1357 |  |
| Mc-0 | N1363 |  |
| Me-0 | N1365 |  |
| Mh-1 | N1369 |  |
| Mir-0 | N1379 |  |
| Mnz-0 | N1371 |  |
| Mr-0 | N1373 |  |
| Mrk-0 | N1375 |  |
| Ms-0 | N1377 |  |
| Mt-0 | N1381 |  |
| Mv-0 | N1387 |  |
| Mz-0 | N1383 |  |
| Na-1 | N1385 |  |
| Nc-1 | N1389 |  |
| Nd-0 | N1391 |  |
| Nd-1 | N1680 |  |
| NFA8 |  | CS22687 |
| NFC10 | N22191 |  |
| NFE1 | N22163 |  |
| NFE10 | N22172 |  |
| Nie-0 | N1393 |  |
| Nok-0 | N1399 |  |
| Nok-1 | N1401 |  |
| Nok-2 | N1403 |  |
| Nok-3 | N1405 |  |
| Np-0 | N1397 |  |
| Nw-0 | N1409 |  |
| Nw-1 | N1411 |  |
| Nw-2 | N1413 |  |
| Nw-3 | N1415 |  |
| Ob-0 | N1419 |  |
| Ob-1 | N1421 |  |
| Ob-2 | N1423 |  |
| Ob-3 | N1425 |  |
| Old-1 | N1427 |  |
| Old-2 | N1429 |  |
| Or-0 | N1433 |  |
| Ost-0 | N1431 |  |
| Ove-0 | N1435 |  |
| Oy-1 | N6929 |  |
| Pa-2 | N1441 |  |
| Pa-3 | N1443 |  |
| Per-2 | N1449 |  |
| Per-3 | N1451 |  |
| Pf-0 | N1453 |  |
| Pi-0 | N1455 |  |
| Pi-2 | N1457 |  |
| Pla-0 | N1459 |  |
| Pla-1 | N1461 |  |
| Pla-2 | N1463 |  |
| Po-1 | N1473 |  |
| Pog-0 | N1477 |  |
| Pr-0 | N1475 |  |
| PUZ16 | N22451 |  |
| Ra-0 | N1481 |  |
| Rd-0 | N1482 |  |
| REN11 | N22263 |  |
| Ri-0 | N1493 |  |
| RLD1 | N913 |  |
| Rou-0 | N1489 |  |
| RP1 | N22362 |  |
| RP10 | N22371 |  |
| RRS10 |  | CS22689 |
| RRS7 |  | CS22688 |
| Rsch-0 | N1491 |  |
| Rsch-4 | N1495 |  |
| Sav-0 | N1515 |  |
| Se-0 | N1503 |  |
| Sf-2 | N1517 |  |
| Sg-1 | N1519 |  |
| Sg-2 | N1521 |  |
| Shahdara | N6180 | CS22690 |
| Shokei | N1676 |  |
| Sp-0 | N1531 |  |
| SQ4 | N22243 |  |
| St-0 | N1535 |  |
| Ste-0 | N1537 |  |
| Stw-0 | N1539 |  |
| Su-0 | N1541 |  |
| Ta-0 | N1549 |  |
| Tamm-2 |  | CS22691 |
| Te-0 | N1551 |  |
| Ts-1 | N1553 |  |
| Ts-5 | N6871 |  |
| Ts-6 | N1561 |  |
| Ts-7 | N1563 |  |
| Tsu-1 |  | CS22693 |
| Tu-1 | N1569 |  |
| Ty-0 | N1573 |  |
| Uk-1 | N1575 |  |
| Uk-10 | New collection | |
| Uk-11 | New collection | |
| Uk-12 | New collection | |
| Uk-13 | New collection | |
| Uk-14 | New collection | |
| Uk-2 | N1579 |  |
| Uk-3 | N1577 |  |
| Uk-4 | N1581 |  |
| Uk-5 | New collection | |
| Uk-6 | New collection | |
| Uk-7 | New collection | |
| Uk-8 | New collection | |
| Uk-9 | New collection | |
| Van-0 | N1585 |  |
| Wa-1 | N1587 |  |
| Wc-1 | N1589 |  |
| Wc-2 | N1591 |  |
| Wei-0 | N6182 |  |
| Wei-1 | N1683 |  |
| Wil-1 | N1595 |  |
| Wil-3 | N1599 |  |
| Wl-0 | N1631 |  |
| Wt-1 | N1605 |  |
| Wt-4 | N1611 |  |
| Wt-5 | N1613 |  |
| Yo-0 | N1622 |  |
| Zü-0 | N1627 |  |

ABRC =	Arabidopsis Biological Resources Center;

NASC =	Nottingham Arabidopsis Stock Centre.
