# Supplementary material for: Autoimmune Response as a Mechanism for a Dobzhansky-Muller-Type Incompatibility Syndrome in Plants
Source: PLoS Biol. 2007 Sep 4;5(9):e236. doi: 10.1371/journal.pbio.0050236 (PMC1964774; doi:10.1371/journal.pbio.0050236)
Supplement: Supplementary file 7 [file pbio.0050236.st004.doc]

**Table S4** Crosses performed and hybrid phenotypes

| **Female** | **Male** | **F_1_ pheno–type** | **Comment** |
| --- | --- | --- | --- |

| 2n Ler | Blh-1 | Normal |  |
| --- | --- | --- | --- |
| 4n Ler | Bla-5 | Normal |  |
| 4n Ler | Wa-1 | Normal |  |
| Aa-0 | Ga-0 | Normal |  |
| Aa-0 | HR15 | Normal |  |
| Aa-0 | Uk-1 | Normal |  |
| Ak-1 | Uk-1 | Normal |  |
| Ak-1 | Uk-3 | Normal |  |
| ANH1 | Gr-1 | Normal |  |
| ANH1 | Kro-0 | Normal |  |
| Ba-1 | Chi-1 | Normal |  |
| Ba-1 | Lov-5 | Normal |  |
| Ba-1 | Shokei | Normal |  |
| Bay-0 | Br-0 | Normal |  |
| Bay-0 | C24 | Normal |  |
| Bay-0 | Col-0 | Normal |  |
| Bay-0 | Fei-0 | Normal |  |
| Bay-0 | Tamm2 | Normal |  |
| Bay-0 | Ts-1 | Normal |  |
| Bch-3 | BG9 | Normal |  |
| Bch-4 | Ei-6 | Normal |  |
| Bch-4 | Pr-0 | Normal |  |
| Bd-0 | Ep-0 | Normal |  |
| Be-1 | Bu-13 | Normal |  |
| Be-1 | Di-2 | Normal |  |
| Be-1 | Ga-2 | Normal |  |
| Be-1 | Hi-0 | Normal |  |
| Be-1 | Nie-0 | Normal |  |
| BG1 | Rsch-0 | Normal |  |
| BG1 | Uk-3 | Normal |  |
| BG2 | BG7 | Normal |  |
| BG2 | Bsch-0 | Normal |  |
| BG5 | Kro-0 | Normal |  |
| BG5 | Ra-0 | Normal |  |
| BG7 | Po-1 | Normal |  |
| Bla-1 | Bla-11 | Normal |  |
| Bla-1 | Bla-12 | Normal |  |
| Bla-1 | Bla14n | Normal |  |
| Bla-1 | Bla-2 | Normal |  |
| Bla-1 | Bla-3 | Normal |  |
| Bla-1 | Bla-4 | Normal |  |
| Bla-1 | Bla-6 | Normal |  |
| **Bla-1** | **Hh-0** | **Class 2 necrosis** | |
| Bla-1 | KZ10 | Normal |  |
| Bla-1 | Mir-0 | Normal |  |
| Bla-1 | Pla-0 | Normal |  |
| Bla-1 | Pla-2 | Normal |  |
| Bla-1 | Ts-5 | Normal |  |
| Bla-1 | Ts-7 | Normal |  |
| Bla-1 | Uk-1 | Normal |  |
| Bla-1 | Uk-3 | Normal |  |
| **Bla-1** | **Sha.** | **Class 2 necrosis** | |
| Bla-11 | Mir-0 | Normal |  |
| Bla-11 | Se-0 | Normal |  |
| Bla-12 | Bla-11 | Normal |  |
| Bla-12 | Bla-14 | Normal |  |
| Bla-12 | Bla-2 | Normal |  |
| Bla-12 | Bla-3 | Normal |  |
| Bla-12 | Hh-0 | Normal |  |
| Bla-12 | Mir-0 | Normal |  |
| Bla-12 | Se-0 | Normal |  |
| Bla-12 | Sha. | Normal |  |
| Bla-12 | Ts-6 | Normal |  |
| Bla-14 | Bla-11 | Normal |  |
| Bla-14 | Bla-2 | Normal |  |
| Bla-14 | Bla-3 | Normal |  |
| Bla-14 | Bla-4 | Normal |  |
| Bla-14 | Ll-0 | Normal |  |
| Bla-14 | Mir-0 | Normal |  |
| Bla-14 | Mt-0 | Normal |  |
| Bla-14 | Pi-2 | Normal |  |
| Bla-14 | Pla-0 | Normal |  |
| Bla-14 | Se-0 | Normal |  |
| Bla-14 | Sha. | Normal |  |
| Bla-14 | Ts-7 | Normal |  |
| Bla14n | Ep-0 | Normal |  |
| Bla14n | Est-0 | Normal |  |
| Bla14n | Ga-0 | Normal |  |
| Bla14p | Per-3 | Normal |  |
| Bla-2 | Bla-11 | Normal |  |
| Bla-2 | Bla-3 | Normal |  |
| Bla-2 | Bla-6 | Normal |  |
| Bla-2 | Ll-11 | Normal |  |
| Bla-2 | Mir-0 | Normal |  |
| Bla-2 | Se-0 | Normal |  |
| **Bla-2** | **Sha.** | **Class 2 necrosis** | |
| Bla-2 | Ts-7 | Normal |  |
| Bla-2 | Uk-1 | Normal |  |
| Bla-2 | Uk-3 | Normal |  |
| Bla-3 | Bla-11 | Normal |  |
| Bla-3 | Bla-4 | Normal |  |
| Bla-3 | Bla-6 | Normal |  |
| Bla-3 | Hh-0 | Normal |  |
| Bla-3 | Ll-11 | Normal |  |
| Bla-3 | Mir-0 | Normal |  |
| Bla-3 | Pla-0 | Normal |  |
| Bla-3 | Pla-2 | Normal |  |
| **Bla-3** | **Se-0** | **Class 1 necrosis** | |
| Bla-3 | Sha. | Normal |  |
| Bla-3 | Ts-5 | Normal |  |
| Bla-3 | Ts-6 | Normal |  |
| Bla-3 | Ts-7 | Normal |  |
| Bla-3 | Uk-1 | Normal |  |
| Bla-3 | Uk-3 | Normal |  |
| Bla-4 | Bla-11 | Normal |  |
| Bla-4 | Bla-12 | Normal |  |
| Bla-4 | Bla-2 | Normal |  |
| Bla-4 | Bla-6 | Normal |  |
| Bla-4 | Hh-0 | Normal |  |
| **Bla-4** | **Ll-0** | **Class 1 necrosis**  **Mild** | |
| Bla-4 | Ll-11 | Normal |  |
| Bla-4 | Mir-0 | Normal |  |
| Bla-4 | Pla-1 | Normal |  |
| Bla-4 | Pla-2 | Normal |  |
| Bla-4 | Se-0 | Normal |  |
| Bla-4 | Shah-dara | Normal |  |
| Bla-5 | 4nLer | Normal | RC |
| Bla-5 | Bla-14 | HSL | Ploidy |
| Bla-5 | Blh-1 | HSL | Ploidy |
| **Bla-5** | **Hh-0** | **Class 2 necrosis**  **(also ploidy)** | |
| Bla-5 | Wa-1 | Normal |  |
| Bla-6 | Bla-11 | Normal |  |
| Bla-6 | Bla-12 | Normal |  |
| Bla-6 | Bla-14 | Normal |  |
| Bla-6 | Mir-0 | Normal |  |
| Bla-6 | Se-0 | Normal |  |
| Bla-6 | Ts-7 | Normal |  |
| Blh-1 | 2nLer | Normal |  |
| Blh-1 | Bla-5 | HSL | Ploidy;RC |
| Blh-1 | Hh-0 | Normal |  |
| Blh-1 | Wa-1 | HSL | Ploidy |
| Blh-2 | Bla-5 | HSL | Ploidy |
| Blh-2 | Blh-1 | Normal |  |
| Bor-4 | Bay-0 | Normal |  |
| Bor-4 | Br-0 | Normal |  |
| Bor-4 | C24 | Normal |  |
| Bor-4 | Cvi-0 | Normal |  |
| Bor-4 | Fei-0 | Normal |  |
| Bor-4 | NFA8 | Normal |  |
| Br-0 | C24 | Normal |  |
| Br-0 | Col-0 | Normal |  |
| Br-0 | Fei-0 | Normal |  |
| Br-0 | St-0 | Normal |  |
| Br-0 | Wil-3 | Normal |  |
| Bs-1 | Bs-2 | Normal |  |
| Bs-1 | Uk-1 | Normal |  |
| Bs-1 | Uk-3 | Normal |  |
| Bs-2 | Lö-2 | Normal |  |
| Bs-2 | Uk-1 | Normal |  |
| Bs-2 | Uk-3 | Normal |  |
| Bs-5 | Bs-2 | Normal |  |
| Bs-5 | Bs-2.2 | Normal |  |
| Bs-5 | Kn-0 | Normal |  |
| Bs-5 | Lö-1 | Normal |  |
| Bs-5 | Lö-2 | Normal |  |
| Bs-5 | Uk-1 | Normal |  |
| Bs-5 | Uk-3 | Normal |  |
| Bsch-0 | HR5 | Normal |  |
| Bsch-0 | HR8 | Normal |  |
| Bsch-0 | NFE10 | Normal |  |
| Bsch-1 | GOT10 | Normal |  |
| Bu-0 | Bsch-2 | Normal |  |
| Bu-0 | NFE10 | Normal |  |
| Bu-11 | Po-1 | Normal |  |
| Bur-0 | Bay-0 | Normal |  |
| Bur-0 | Br-0 | Normal |  |
| Bur-0 | C24 | Normal |  |
| Bur-0 | Col-0 | Normal |  |
| Bur-0 | Est-1 | Normal |  |
| Bur-0 | Lov-5 | Normal |  |
| Bur-0 | Pf-0 | Normal |  |
| Bur-0 | Ts-1 | Normal |  |
| Ca-0 | Bu-11 | Normal |  |
| Cal-0 | Bur-0 | Normal |  |
| Cal-0 | Ei-5 | Normal |  |
| Cal-0 | Lm-2 | Normal |  |
| Cal-0 | Wil-1 | Normal |  |
| Can-0 | Bla-1 | Normal |  |
| Can-0 | Mt-0 | Normal |  |
| Can-0 | St-0 | Normal |  |
| Cen-0 | Dr-0 | Normal |  |
| Cen-0 | Hl-2 | Normal |  |
| Cen-0 | Ost-0 | Normal |  |
| Cen-0 | Se-0 | Normal |  |
| Cha-0 | Pla-0 | Normal |  |
| Cha-0 | Se-0 | Normal |  |
| Chi-0 | Ak-1 | Normal |  |
| Chi-0 | Bla-1 | Normal |  |
| Chi-0 | Uk-1 | Normal |  |
| Chi-0 | Uk-3 | Normal |  |
| Chi-2 | Chi-0 | Normal |  |
| Chi-2 | KZ10 | Normal |  |
| CIBC1 | Bu-11 | Normal |  |
| CIBC1 | Chi-1 | Normal |  |
| CIBC10 | Bl-1 | Normal |  |
| CIBC10 | Hodja-Obi | Normal |  |
| Cit-0 | CIBC10 | Normal |  |
| Cit-0 | Ei-6 | Normal |  |
| Cit-0 | Gy-0 | Normal |  |
| Cnt-1 | Gr-1 | Normal |  |
| Co-1 | Co-3 | Normal |  |
| Co-1 | Oy-1 | Normal |  |
| Co-3 | Te-0 | Normal |  |
| Co-4 | Co-3 | Normal |  |
| Co-4 | Wil-1 | Normal |  |
| Col-0 | Bla-5 | HSL | Ploidy |
| Col-0 | Blh-1 | Normal |  |
| Col-0 | C24 | Normal |  |
| Col-0 | Chi-2 | Normal |  |
| Col-0 | Fei-0 | Normal |  |
| Col-0 | GOT7 | Normal |  |
| Col-0 | NFA8 | Normal |  |
| Col-0 | Nie-0 | Normal |  |
| Col-0 | Tamm2 | Normal |  |
| Col-0 | Ts-1 | Normal |  |
| Ct-1 | St-0 | Normal |  |
| Cvi-0 | Bur-0 | Normal |  |
| Cvi-0 | RRS7 | Normal |  |
| Cvi-0 | Uk-1 | Normal |  |
| Cvi-0 | Uk-3 | Normal |  |
| Db-1 | Db-0 | Normal |  |
| Di-2 | Ra-0 | Normal |  |
| Dra-1 | Dra-0 | Normal |  |
| Edi-0 | En-2 | Normal |  |
| Ei-2 | Lm-2 | Normal |  |
| Ei-2 | Na-1 | Normal |  |
| Ei-2 | Ove-0 | Normal |  |
| Ei-4 | CIBC1 | Normal |  |
| Ei-5 | Ei-2 | Normal |  |
| Ei-5 | Oy-1 | Normal |  |
| Eil-0 | El-0 | Normal |  |
| El-0 | Eil-0 | Normal | RC |
| Ema-1 | Estland | Normal |  |
| En-1 | Uk-3 | Normal |  |
| En-2 | RLD1 | Normal |  |
| Er-0 | Ang-1 | Normal |  |
| Er-0 | Dra-1 | Normal |  |
| Est-0 | Hau-0 | Normal |  |
| Est-0 | Wa-1 | HSL | Ploidy |
| Est-1 | Br-0 | Normal |  |
| Est-1 | Cvi-0 | Normal |  |
| Est-1 | Est-0 | Normal |  |
| Est-1 | Fei-0 | Normal |  |
| Est-1 | Mt-0 | Normal |  |
| Est-1 | RRS10 | Normal |  |
| Est-1 | Ts-1 | Normal |  |
| Et-0 | Bla-1 | Normal |  |
| Et-0 | Fe-1 | Normal |  |
| Fe-1 | Ge-2 | Normal |  |
| Fe-1 | Uk-1 | Normal |  |
| Fe-1 | Uk-3 | Normal |  |
| Fei-0 | NFA8 | Normal |  |
| Fei-0 | Ts-1 | Normal |  |
| Fi-0 | Gr-3 | Normal |  |
| FM11 | KZ10 | Normal |  |
| FM11 | Pa-2 | Normal |  |
| Fr-3 | Hodja-Obi | Normal |  |
| Fr-4 | Fr-2 | Normal |  |
| Fr-5 | Fr-4 | Normal |  |
| Fr-6 | RLD1 | Normal |  |
| Fr-6 | Uk-1 | Normal |  |
| Fr-6 | Uk-3 | Normal |  |
| Fr-7 | ENK-T | Normal |  |
| Fr-7 | Uk-1 | Normal |  |
| Fr-7 | Uk-3 | Normal |  |
| Gd-1 | BG5 | Normal |  |
| Gd-1 | Bu-13 | Normal |  |
| Gd-1 | NFC10 | Normal |  |
| Gd-1 | Ste-0 | Normal |  |
| Ge-1 | Li-2:1 | Normal |  |
| Ge-2 | Cha-0 | Normal |  |
| Gie-0 | Fi-1 | Normal |  |
| Gö-0 | Gö-2 | Normal |  |
| GOT1 | Chi-2 | Normal |  |
| GOT1 | Gr-2 | Normal |  |
| GOT1 | Gy-0 | Normal |  |
| GOT1 | Nok-3 | Normal |  |
| GOT7 | Bur-0 | Normal |  |
| GOT7 | C24 | Normal |  |
| GOT7 | Cvi-0 | Normal |  |
| GOT7 | Est-1 | Normal |  |
| GOT7 | Sha. | Normal |  |
| GOT7 | Ts-1 | Normal |  |
| Gr-1 | M7943S | Normal |  |
| Gr-2 | Can-0 | Normal |  |
| Gr-2 | GOT1 | Normal | RC |
| Gr-2 | Gr-6 | Normal |  |
| Gr-2 | In-0 | Normal |  |
| Gr-3 | Fi-0 | Normal | RC |
| Gr-3 | Gr-5 | Normal |  |
| Gr-3 | Gr-6 | Normal |  |
| Gr-3 | Per-3 | Normal |  |
| Gr-6 | Co-4 | Normal |  |
| Gr-6 | Gr-3 | Normal | RC |
| Gr-6 | Hl-0 | Normal |  |
| Gr-6 | Oy-1 | Normal |  |
| Gr-6 | Rsch-4 | Normal |  |
| Gr-6 | Wa-1 | HSL | Ploidy |
| Gre-0 | Bla-5 | HSL | Ploidy |
| Gre-0 | Chi-0 | Normal |  |
| Gü-0 | Gü-1 | Normal |  |
| Ha-0 | Db-2 | Normal |  |
| Hau-0 | Est-0 | Normal | RC |
| Hau-0 | Sha. | Normal |  |
| Hau-0 | Wa-1 | HSL | Ploidy |
| **Hh-0** | **Bla-1** | **Class 2 necrosis**; RC | |
| Hh-0 | Bla-11 | Normal |  |
| Hh-0 | Bla-14 | Normal |  |
| **Hh-0** | **Bla-2** | **Class 2 necrosis** | |
| Hh-0 | Bla-6 | Normal |  |
| Hh-0 | Col-0 | Normal |  |
| Hh-0 | Mir-0 | Normal |  |
| Hh-0 | Nc-1 | Normal |  |
| **Hh-0** | **Se-0** | **Class 1 necrosis** | |
| Hh-0 | Ts-7 | Normal |  |
| Hh-0 | Uk-1 | Normal |  |
| Hh-0 | Uk-3 | Normal |  |
| Hi-0 | Be-1 | Normal | RC |
| Hi-0 | Pr-0 | Normal |  |
| Hl-0 | Bla-1 | Normal |  |
| Hl-0 | Gie-0 | Normal |  |
| Hl-0 | Pi-0 | Normal |  |
| Hl-2 | Et-0 | Normal |  |
| Hl-2 | Mt-0 | Normal |  |
| Hl-3 | Sha. | Normal |  |
| Hl-3 | Wa-1 | HSL | Ploidy |
| Hn-0 | Bch-4 | Normal |  |
| Hn-0 | Ra-0 | Normal |  |
| Hodja-Obi | Bu-13 | Normal |  |
| Hodja-Obi | Mz-0 | Normal |  |
| HR15 | Aa-0 | Normal | RC |
| HR15 | Bs-1 | Normal |  |
| HR15 | Gr-6 | Normal |  |
| HR15 | Te-0 | Normal |  |
| HR5 | Bu-13 | Normal |  |
| Hs-0 | Hl-0 | Normal |  |
| Hs-1 | ENK-T | Normal |  |
| Hs-1 | Mz-0 | Normal |  |
| Is-0 | Is-1 | Normal |  |
| Is-1 | Nd-0 | Normal |  |
| Je-0 | Gie-0 | Normal |  |
| Je-0 | Pa-3 | Normal |  |
| Jl-1 | Eil-0 | Normal |  |
| Jl-1 | Sha. | Normal |  |
| Jl-1 | Uk-1 | Normal |  |
| Jl-1 | Uk-3 | Normal |  |
| Jl-2 | Aa-0 | Normal |  |
| Jl-2 | Jl-1 | Normal |  |
| Jl-2 | Mt-0 | Normal |  |
| Jl-2 | Uk-1 | Normal |  |
| Jl-2 | Uk-3 | Normal |  |
| Js-0 | Bu-13 | Normal |  |
| Kb-0 | M7943S | Normal |  |
| Kil-0 | An-1 | Normal |  |
| Kl-0 | Kl-1 | Normal |  |
| Kn-0 | Uk-3 | Normal |  |
| KNO1 | M7943S | Normal |  |
| Kondara | NFE1 | Normal |  |
| Kondara | YGAP1 | Normal |  |
| Kro-0 | Sf-2 | Normal |  |
| KZ10 | Gie-0 | Normal |  |
| KZ10 | Hh-0 | Normal |  |
| KZ10 | Mir-0 | Normal |  |
| **KZ10** | **Mrk-0** | **Class 3 necrosis** | |
| KZ10 | Uk-1 | Normal |  |
| KZ10 | Uk-3 | Normal |  |
| KZ11 | Ei-4 | Normal |  |
| KZ11 | HR14 | Normal |  |
| **KZ11** | **Mrk-0** | **Class 3 necrosis** | |
| KZ11 | NFC1 | Normal |  |
| KZ11 | Ob-0 | Normal |  |
| KZ11 | YGAP1 | Normal |  |
| La-1 | ENK-T | Normal |  |
| La-1 | Fr-6 | Normal |  |
| La-1 | YGAP1 | Normal |  |
| Lan-0 | Ll-2 | Normal |  |
| Le-0 | GOT1 | Normal |  |
| Le-0 | Ll-0 | Normal |  |
| Le-0 | Ost-0 | Normal |  |
| Le-0 | Pf-0 | Normal |  |
| Ler-1 | Br-0 | Normal |  |
| Ler-1 | Fei-0 | Normal |  |
| Ler-1 | Ts-1 | Normal |  |
| Ler-1 | Van-0 | Normal |  |
| Li-10 | Kin-0 | Normal |  |
| Li-2 | Hodja-Obi | Normal |  |
| Li-2 | HR5 | Normal |  |
| Li-2:1 | Bla-5 | HSL | Ploidy |
| Li-2:1 | Chi-2 | Normal |  |
| Li-2:1 | Wa-1 | HSL | Ploidy |
| Li-2:1 | Wil-3 | Normal |  |
| Lip-0 | Ei-6 | Normal |  |
| Lip-0 | Kn-0 | Normal |  |
| Lip-0 | Uk-1 | Normal |  |
| Lip-0 | Uk-3 | Normal |  |
| Ll-0 | Bla-1 | Normal |  |
| Ll-0 | Bla-11 | Normal |  |
| Ll-0 | Bla-12 | Normal |  |
| Ll-0 | Bla-2 | Normal |  |
| Ll-0 | Bla-3 | Normal |  |
| Ll-0 | Bla-6 | Normal |  |
| Ll-0 | Br-0 | Normal |  |
| Ll-0 | Fr-2 | Normal |  |
| Ll-0 | Hh-0 | Normal |  |
| Ll-0 | Ll-11 | Normal |  |
| Ll-0 | Ll-2 | Normal |  |
| Ll-0 | Mir-0 | Normal |  |
| Ll-0 | Se-0 | Normal |  |
| Ll-0 | Ts-5 | Normal |  |
| Ll-0 | Ts-7 | Normal |  |
| Ll-11 | Bla-1 | Normal |  |
| Ll-11 | Bla-11 | Normal |  |
| Ll-11 | Bla-12 | Normal |  |
| Ll-11 | Bla-14 | Normal |  |
| Ll-11 | Bla-6 | Normal |  |
| Ll-11 | Cha-0 | Normal |  |
| Ll-11 | Hh-0 | Normal |  |
| Ll-11 | Ll-0 | Normal | RC |
| Ll-11 | Mir-0 | Normal |  |
| Ll-11 | Ove-0 | Normal |  |
| Ll-11 | Ts-1 | Normal |  |
| **Ll-11** | **Ts-6** | **Class 2 necrosis**  **(transient)** | |
| Ll-11 | Ts-7 | Normal |  |
| Ll-2 | Bla-1 | Normal |  |
| Ll-2 | Bla-11 | Normal |  |
| Ll-2 | Bla-12 | Normal |  |
| Ll-2 | Bla-14 | Normal |  |
| Ll-2 | Bla-2 | Normal |  |
| Ll-2 | Bla-3 | Normal |  |
| Ll-2 | Bla-4 | Normal |  |
| Ll-2 | Bla-6 | Normal |  |
| Ll-2 | Hh-0 | Normal |  |
| Ll-2 | Ll-11 | Normal |  |
| Ll-2 | Mir-0 | Normal |  |
| Ll-2 | Se-0 | Normal |  |
| Ll-2 | Ts-1 | Normal |  |
| Ll-2 | Ts-5 | Normal |  |
| Ll-2 | Ts-7 | Normal |  |
| Lm-2 | Ei-2 | Normal | RC |
| Lö-1 | Bs-1 | Normal |  |
| Lö-1 | Bs-2 | Normal |  |
| Lö-1 | Ost-0 | Normal |  |
| Lö-1 | Uk-1 | Normal |  |
| Lö-1 | Uk-3 | Normal |  |
| Lö-1 | Uk-4 | Normal |  |
| Lö-2 | Bs-1 | Normal |  |
| Lö-2 | Lö-1 | Normal |  |
| Lö-2 | Shokei | Normal |  |
| Lö-2 | Uk-1 | Normal |  |
| Lö-2 | Uk-3 | Normal |  |
| Lö-2 | Yo-0 | Normal |  |
| Lov-5 | Bay-0 | Normal |  |
| Lov-5 | Col-0 | Normal |  |
| Lov-5 | Est-1 | Normal |  |
| Lov-5 | Fei-0 | Normal |  |
| Lov-5 | GOT7 | Normal |  |
| Lov-5 | Ts-1 | Normal |  |
| Ma-0 | Jm-1 | Normal |  |
| Me-0 | Hn-0 | Normal |  |
| Me-0 | Kro-0 | Normal |  |
| Mh-1 | M78845 | Normal |  |
| Mir-0 | Hau-0 | Normal |  |
| **Mir-0** | **Se-0** | **Class 1 necrosis** | |
| Mir-0 | Uk-3 | Normal |  |
| Mir-0 | Wil-3 | Normal |  |
| Mnz-0 | BG7 | Normal |  |
| Mnz-0 | Hl-0 | Normal |  |
| Mrk-0 | Bla-1 | Normal |  |
| Mrk-0 | Bla-1 | Normal |  |
| Mrk-0 | Co-4 | Normal |  |
| Mrk-0 | Hh-0 | Normal |  |
| **Mrk-0** | **KZ10** | **Class 3 necrosis**; RC | |
| Mrk-0 | Mir-0 | Normal |  |
| Mrk-0 | Sha. | Normal |  |
| Mrk-0 | Uk-1 | Normal |  |
| Mrk-0 | Uk-3 | Normal |  |
| Ms-0 | Lm-2 | Normal |  |
| Ms-0 | Pa-2 | Normal |  |
| Mt-0 | Aa-0 | Normal |  |
| Mt-0 | Jl-2 | Normal | RC |
| Mt-0 | Nw-2 | Normal |  |
| Mt-0 | Pi-2 | Normal |  |
| Mt-0 | Uk-1 | Normal |  |
| Mv-0 | Br-0 | Normal |  |
| Mv-0 | Uk-2 | Normal |  |
| Mz-0 | BG5 | Normal |  |
| Mz-0 | Bu-13 | Normal |  |
| Na-1 | Hl-3 | Normal |  |
| Na-1 | Ove-0 | Normal |  |
| Na-1 | Uk-1 | Normal |  |
| Na-1 | Uk-3 | Normal |  |
| Nd-0 | Nd-1 | Normal |  |
| Nd-0 | Uk-1 | Normal |  |
| NFA8 | Br-0 | Normal |  |
| NFA8 | Est-1 | Normal |  |
| NFA8 | Ts-1 | Normal |  |
| NFA8 | Van-0 | Normal |  |
| NFC1 | Be-1 | Normal |  |
| NFC1 | FM11 | Normal |  |
| NFC1 | Hh-0 | Normal |  |
| NFC1 | Np-0 | Normal |  |
| NFC10 | Kb-0 | Normal |  |
| NFE10 | Me-0 | Normal |  |
| Nie-0 | BG5 | Normal |  |
| Nie-0 | Cen-0 | Normal |  |
| Nie-0 | Chi-2 | Normal |  |
| Nie-0 | Fr-6 | Normal |  |
| Nie-0 | HR5 | Normal |  |
| Nie-0 | Li-10 | Normal |  |
| Nie-0 | Sp-0 | Normal |  |
| Nok-0 | Nok-1 | Normal |  |
| Nok-0 | Uk-1 | Normal |  |
| Nok-0 | Uk-3 | Normal |  |
| Nok-2 | Nok-0 | Normal |  |
| Nok-2 | Nok-1 | Normal |  |
| Nok-2 | Nok-3 | Normal |  |
| Nok-2 | Pla-0 | Normal |  |
| Nok-3 | Nok-0 | Normal |  |
| Nok-3 | Nok-1 | Normal |  |
| Nw-1 | Nw-0 | Normal |  |
| Nw-1 | Uk-1 | Normal |  |
| Nw-1 | Uk-3 | Normal |  |
| Nw-2 | Aa-0 | Normal |  |
| Nw-2 | Jl-2 | Normal |  |
| Nw-2 | Mt-0 | Normal | RC |
| Nw-2 | Nw-3 | Normal |  |
| Nw-2 | Uk-1 | Normal |  |
| Nw-2 | Uk-3 | Normal |  |
| Ob-0 | BG5 | Normal |  |
| Ob-0 | Chi-1 | Normal |  |
| Ob-0 | Hodja-Obi | Normal |  |
| Ob-1 | En-1 | Normal |  |
| Ob-2 | Ob-1 | Normal |  |
| Old-1 | Fr-6 | Normal |  |
| Old-2 | Db-2 | Normal |  |
| Or-0 | Gy-0 | Normal |  |
| Ost-0 | Chi-2 | Normal |  |
| Ost-0 | Per-2 | Normal |  |
| Ove-0 | Lm-2 | Normal |  |
| Oy-1 | Nok-0 | Normal |  |
| Oy-1 | Pla-2 | Normal |  |
| Oy-1 | Uk-1 | Normal |  |
| Oy-1 | Uk-3 | Normal |  |
| Pa-2 | Pa-3 | Normal |  |
| Per-2 | NFC1 | Normal |  |
| Per-2 | Uk-1 | Normal |  |
| Per-3 | Gr-3 | Normal | RC |
| Per-3 | Pi-2 | Normal |  |
| Per-3 | Rd-0 | Normal |  |
| Pf-0 | Uk-1 | Normal |  |
| Pf-0 | Uk-3 | Normal |  |
| Pi-0 | Bur-0 | Normal |  |
| Pla-0 | Bla-1 | Normal | RC |
| Pla-0 | Bla-11 | Normal |  |
| Pla-0 | Bla-12 | Normal |  |
| Pla-0 | Bla-2 | Normal |  |
| Pla-0 | Bla-4 | Normal |  |
| Pla-0 | Bla-6 | Normal |  |
| Pla-0 | Ge-2 | Normal |  |
| Pla-0 | Hh-0 | Normal |  |
| Pla-0 | Ll-0 | Normal |  |
| Pla-0 | Ll-11 | Normal |  |
| Pla-0 | Ll-2 | Normal |  |
| Pla-0 | Mir-0 | Normal |  |
| Pla-0 | Pla-1 | Normal |  |
| Pla-0 | Se-0 | Normal |  |
| Pla-0 | Sha. | Normal |  |
| Pla-0 | Ts-5 | Normal |  |
| Pla-0 | Ts-6 | Normal |  |
| Pla-1 | Bla-1 | Normal |  |
| Pla-1 | Bla-11 | Normal |  |
| Pla-1 | Bla-12 | Normal |  |
| Pla-1 | Bla-14 | Normal |  |
| Pla-1 | Bla-2 | Normal |  |
| **Pla-1** | **Bla-3** | **Class 1 necrosis** | |
| Pla-1 | Bla-6 | Normal |  |
| **Pla-1** | **Hh-0** | **Class 1 necrosis** | |
| Pla-1 | Ll-0 | Normal |  |
| Pla-1 | Ll-11 | Normal |  |
| Pla-1 | Ll-2 | Normal |  |
| **Pla-1** | **Mir-0** | **Class 1 necrosis** | |
| Pla-1 | Pla-2 | Normal |  |
| Pla-1 | Se-0 | Normal |  |
| Pla-1 | Ts-7 | Normal |  |
| Pla-2 | Bla-11 | Normal |  |
| Pla-2 | Bla-12 | Normal |  |
| Pla-2 | Bla-14 | Normal |  |
| Pla-2 | Bla-2 | Normal |  |
| Pla-2 | Bla-6 | Normal |  |
| Pla-2 | Hh-0 | Normal |  |
| Pla-2 | Ll-0 | Normal |  |
| Pla-2 | Ll-11 | Normal |  |
| Pla-2 | Ll-2 | Normal |  |
| Pla-2 | Mir-0 | Normal |  |
| Pla-2 | Pla-0 | Normal |  |
| Pla-2 | Sha. | Normal |  |
| Pla-2 | Ts-7 | Normal |  |
| Po-1 | Wei-0 | Normal |  |
| Pog-0 | Uk-1 | Normal |  |
| Pog-0 | Uk-3 | Normal |  |
| PUZ16 | CSHL1 | Normal |  |
| PUZ16 | Fr-2 | Normal |  |
| PUZ16 | Pog-0 | Normal |  |
| Rd-0 | Rou-0 | Normal |  |
| Rd-0 | Uk-1 | Normal |  |
| **REN11** | **Wei-1** | **Class 3 necrosis** | |
| Ri-0 | Rd-0 | Normal |  |
| RLD1 | NFC10 | Normal |  |
| RLD2 | Me-0 | Normal |  |
| Rou-0 | Rd-0 | Normal | RC |
| Rou-0 | Wt-5 | Normal |  |
| RP1 | Blh-2 | Normal |  |
| RP1 | RP10 | Normal |  |
| RP-1 | Mv-0 | Normal |  |
| RP10 | Blh-1 | Normal |  |
| RP10 | Fe-1 | Normal |  |
| RRS10 | Bor-4 | Normal |  |
| RRS10 | Br-0 | Normal |  |
| RRS10 | Col-0 | Normal |  |
| RRS10 | Fei-0 | Normal |  |
| RRS10 | NFA8 | Normal |  |
| RRS10 | RRS7 | Normal |  |
| RRS10 | Ts-1 | Normal |  |
| RRS7 | Bur-0 | Normal |  |
| RRS7 | Bay-0 | Normal |  |
| RRS7 | Col-0 | Normal |  |
| RRS7 | Est-1 | Normal |  |
| RRS7 | Tamm2 | Normal |  |
| Rsch-0 | BG1 | Normal | RC |
| Rsch-0 | Pog-0 | Normal |  |
| Rsch-4 | Wl-0 | Normal |  |
| Se-0 | Bla-1 | Normal |  |
| Se-0 | KZ10 | Normal |  |
| **Se-0** | **Mir-0** | **Class 1 necrosis**; RC | |
| Se-0 | Mrk-0 | Normal |  |
| Se-0 | Uk-3 | Normal |  |
| Sg-1 | Bs-1 | Normal |  |
| Sg-1 | Sg-2 | Normal |  |
| Sg-1 | Uk-1 | Normal |  |
| Sg-1 | Uk-3 | Normal |  |
| Sg-2 | Lö-2 | Normal |  |
| Sg-2 | Sg-1 | Normal |  |
| Sg-2 | Uk-1 | Normal |  |
| Sg-2 | Uk-3 | Normal |  |
| **Sha.** | **Bla-1** | **Class 2 necrosis**; RC | |
| Sha. | Bla-11 | Normal |  |
| Sha. | Bla-6 | Normal |  |
| Sha. | Br-0 | Normal |  |
| Sha. | C24 | Normal |  |
| Sha. | Col-0 | Normal |  |
| Sha. | Est-1 | Normal |  |
| Sha. | Fei-0 | Normal |  |
| Sha. | Hh-0 | Normal |  |
| Sha. | KZ10 | Normal |  |
| **Sha.** | **Ll-0** | **Class 2 necrosis; mild** | |
| Sha. | Ll-11 | Normal |  |
| Sha. | Ll-2 | Normal |  |
| Sha. | Lov-5 | Normal |  |
| Sha. | Mir-0 | Normal |  |
| Sha. | Pla-1 | Normal |  |
| Sha. | RRS7 | Normal |  |
| Sha. | Se-0 | Normal |  |
| Sha. | Ts-7 | Normal |  |
| Sha. | Uk-1 | Normal |  |
| Sha. | Uk-3 | Normal |  |
| Sha. | Hl-3 | Normal | RC |
| Sha. | Kondara | Normal |  |
| Sp-0 | Gr-1 | Normal |  |
| Sp-0 | Li-10 | Normal |  |
| SQ4 | Uk-2 | Normal |  |
| Ste-0 | Bu-0 | Normal |  |
| Ste-0 | Sf-2 | Normal |  |
| Stw-0 | Jl-1 | Normal |  |
| Su-0 | Ak-1 | Normal |  |
| Su-0 | Blh-1 | Normal |  |
| Su-0 | Chi-0 | Normal |  |
| Su-0 | Col-0 | Normal |  |
| Su-0 | Hh-0 | Normal |  |
| Su-0 | Tsu-1 | Normal |  |
| Ta-0 | SQ4 | Normal |  |
| Tamm-2 | Bor-4 | Normal |  |
| Tamm-2 | Br-0 | Normal |  |
| Tamm-2 | Cvi-0 | Normal |  |
| Tamm-2 | Est-1 | Normal |  |
| Tamm-2 | Fei-0 | Normal |  |
| Tamm-2 | Lov-5 | Normal |  |
| Tamm-2 | RRS10 | Normal |  |
| Tamm-2 | Ts-1 | Normal |  |
| Te-0 | Bla-1 | Normal |  |
| Te-0 | Je-0 | Normal |  |
| Te-0 | Pf-0 | Normal |  |
| Te-0 | Uk-1 | Normal |  |
| Te-0 | Uk-3 | Normal |  |
| Te-0 | Wil-1 | Normal |  |
| Ts-1 | Bla-1 | Normal |  |
| Ts-1 | Bla-11 | Normal |  |
| Ts-1 | Bla-12 | Normal |  |
| Ts-1 | Bla-14 | Normal |  |
| Ts-1 | Bla-2 | Normal |  |
| Ts-1 | Bla-3 | Normal |  |
| Ts-1 | Bla-4 | Normal |  |
| Ts-1 | Bla-6 | Normal |  |
| Ts-1 | Cvi-0 | Normal |  |
| Ts-1 | Hh-0 | Normal |  |
| Ts-1 | Ll-0 | Normal |  |
| Ts-1 | Mir-0 | Normal |  |
| Ts-1 | Pla-0 | Normal |  |
| Ts-1 | Pla-1 | Normal |  |
| Ts-1 | Pla-2 | Normal |  |
| Ts-1 | Se-0 | Normal |  |
| **Ts-1** | **Sha.** | **Class 2 necrosis (transient); RC** | |
| Ts-1 | Ts-7 | Normal |  |
| Ts-1 | Uk-1 | Normal |  |
| Ts-1 | Uk-3 | Normal |  |
| Ts-5 | Bla-11 | Normal |  |
| Ts-5 | Bla-12 | Normal |  |
| Ts-5 | Bla-14 | Normal |  |
| Ts-5 | Bla-2 | Normal |  |
| Ts-5 | Bla-4 | Normal |  |
| Ts-5 | Bla-6 | Normal |  |
| Ts-5 | Hh-0 | Normal |  |
| Ts-5 | Ll-0 | Normal | RC |
| Ts-5 | Ll-11 | Normal |  |
| Ts-5 | Mir-0 | Normal |  |
| Ts-5 | Pla-1 | Normal |  |
| Ts-5 | Pla-2 | Normal |  |
| Ts-5 | Se-0 | Normal |  |
| Ts-5 | Sha. | Normal |  |
| Ts-5 | Ts-1 | Normal |  |
| Ts-5 | Ts-6 | Normal |  |
| Ts-5 | Ts-7 | Normal |  |
| Ts-5 | Uk-1 | Normal |  |
| Ts-6 | Bla-1 | Normal |  |
| Ts-6 | Bla-11 | Normal |  |
| Ts-6 | Bla-12 | Normal | RC |
| Ts-6 | Bla-14 | Normal |  |
| Ts-6 | Bla-2 | Normal |  |
| Ts-6 | Bla-4 | Normal |  |
| Ts-6 | Bla-6 | Normal |  |
| Ts-6 | Hh-0 | Normal |  |
| Ts-6 | Ll-0 | Normal |  |
| Ts-6 | Ll-2 | Normal |  |
| Ts-6 | Mir-0 | Normal |  |
| Ts-6 | Pla-1 | Normal |  |
| Ts-6 | Pla-2 | Normal |  |
| Ts-6 | Se-0 | Normal |  |
| Ts-6 | Sha. | Normal |  |
| Ts-6 | Ts-1 | Normal |  |
| Ts-6 | Ts-7 | Normal |  |
| Ts-6 | Uk-1 | Normal |  |
| Ts-6 | Uk-3 | Normal |  |
| Ts-7 | Bla-1 | Normal | RC |
| Ts-7 | Bla-12 | Normal |  |
| Ts-7 | Bla-4 | Normal |  |
| Ts-7 | Hh-0 | Normal | RC |
| Ts-7 | Mir-0 | Normal |  |
| Ts-7 | Nw-1 | Normal |  |
| Ts-7 | Pa-3 | Normal |  |
| Ts-7 | Pla-0 | Normal |  |
| Ts-7 | Se-0 | Normal |  |
| Tsu-1 | Bay-0 | Normal |  |
| Tsu-1 | C24 | Normal |  |
| Tsu-1 | Col-0 | Normal |  |
| Tsu-1 | Est-1 | Normal |  |
| Tsu-1 | RRS10 | Normal |  |
| Tsu-1 | Tamm2 | Normal |  |
| Tu-1 | Pa-3 | Normal |  |
| Tu-1 | Ts-7 | Normal |  |
| Ty-0 | Mc-0 | Normal |  |
| Ty-0 | NFC1 | Normal |  |
| Ty-0 | Tsu-1 | Normal |  |
| Uk-1 | Bd-0 | Normal |  |
| Uk-1 | BG1 | Normal |  |
| Uk-1 | Col-0 | Normal |  |
| Uk-1 | En-1 | Normal |  |
| Uk-1 | Er-0 | Normal |  |
| Uk-1 | Kn-0 | Normal |  |
| Uk-1 | Mir-0 | Normal |  |
| **Uk-1** | **Nc-1** | **Class 3 necrosis** | |
| Uk-1 | Np-0 | Normal |  |
| Uk-1 | Se-0 | Normal |  |
| Uk-1 | Uk-10 | Normal |  |
| Uk-1 | Uk-11 | Normal |  |
| Uk-1 | Uk-12 | Normal |  |
| Uk-1 | Uk-13 | Normal |  |
| Uk-1 | Uk-2 | Normal |  |
| **Uk-1** | **Uk-3** | **Class 3 necrosis** | |
| Uk-1 | Uk-5 | Normal |  |
| Uk-1 | Uk-7 | Normal |  |
| Uk-1 | Uk-8 | Normal |  |
| Uk-1 | Uk-9 | Normal |  |
| Uk-1 | Wt-4 | Normal |  |
| Uk-10 | Uk-11 | Normal |  |
| Uk-10 | Uk-9 | Normal |  |
| Uk-11 | Uk-12 | Normal |  |
| Uk-11 | Uk-14 | Normal |  |
| Uk-11 | Uk-5 | Normal |  |
| Uk-11 | Uk-7 | Normal |  |
| Uk-11 | Uk-9 | Normal |  |
| Uk-13 | Uk-10 | Normal |  |
| Uk-13 | Uk-11 | Normal |  |
| Uk-13 | Uk-12 | Normal |  |
| Uk-13 | Uk-7 | Normal |  |
| Uk-14 | Uk-1 | Normal |  |
| Uk-14 | Uk-10 | Normal |  |
| Uk-14 | Uk-12 | Normal |  |
| Uk-14 | Uk-13 | Normal |  |
| Uk-14 | Uk-7 | Normal |  |
| Uk-3 | Aa-0 | Normal |  |
| Uk-3 | Bd-0 | Normal |  |
| Uk-3 | Col-0 | Normal |  |
| Uk-3 | Er-0 | Normal |  |
| Uk-3 | Mt-0 | Normal |  |
| Uk-3 | Nc-1 | Normal |  |
| Uk-3 | Np-0 | Normal |  |
| **Uk-3** | **Uk-1** | **Class 3 necrosis**; RC | |
| Uk-3 | Uk-10 | Normal |  |
| Uk-3 | Uk-11 | Normal |  |
| Uk-3 | Uk-12 | Normal |  |
| Uk-3 | Uk-13 | Normal |  |
| Uk-3 | Uk-14 | Normal |  |
| Uk-3 | Uk-2 | Normal |  |
| Uk-3 | Uk-5 | Normal |  |
| Uk-3 | Uk-6 | Normal |  |
| Uk-3 | Uk-7 | Normal |  |
| Uk-3 | Uk-8 | Normal |  |
| Uk-3 | Uk-9 | Normal |  |
| Uk-3 | Wt-4 | Normal |  |
| Uk-4 | Pa-3 | Normal |  |
| Uk-4 | Uk-1 | Normal |  |
| Uk-4 | Uk-3 | Normal |  |
| Uk-5 | Uk-10 | Normal |  |
| Uk-5 | Uk-12 | Normal |  |
| Uk-5 | Uk-13 | Normal |  |
| Uk-5 | Uk-14 | Normal |  |
| Uk-5 | Uk-6 | Normal |  |
| Uk-5 | Uk-9 | Normal |  |
| **Uk-6** | **Uk-1** | **Class 3 necrosis** | |
| Uk-6 | Uk-10 | Normal |  |
| Uk-6 | Uk-11 | Normal |  |
| Uk-6 | Uk-12 | Normal |  |
| Uk-6 | Uk-13 | Normal |  |
| Uk-6 | Uk-14 | Normal |  |
| Uk-6 | Uk-7 | Normal |  |
| Uk-6 | Uk-8 | Normal |  |
| Uk-6 | Uk-9 | Normal |  |
| Uk-7 | Uk-10 | Normal |  |
| Uk-7 | Uk-12 | Normal |  |
| Uk-7 | Uk-5 | Normal |  |
| Uk-7 | Uk-8 | Normal |  |
| Uk-7 | Uk-9 | Normal |  |
| Uk-8 | Uk-10 | Normal |  |
| Uk-8 | Uk-11 | Normal |  |
| Uk-8 | Uk-12 | Normal |  |
| Uk-8 | Uk-13 | Normal |  |
| Uk-8 | Uk-14 | Normal |  |
| Uk-8 | Uk-5 | Normal |  |
| Uk-8 | Uk-9 | Normal |  |
| Uk-9 | Uk-12 | Normal |  |
| Uk-9 | Uk-13 | Normal |  |
| Uk-9 | Uk-14 | Normal |  |
| Van-0 | Bay-0 | Normal |  |
| Van-0 | Bor-4 | Normal |  |
| Van-0 | Br-0 | Normal |  |
| Van-0 | C24 | Normal |  |
| Van-0 | Fei-0 | Normal |  |
| Van-0 | Ts-1 | Normal |  |
| Wa-1 | 4nLer | Normal |  |
| Wa-1 | Bla-5 | Normal |  |
| Wa-1 | Blh-1 | HSL | Ploidy |
| Wa-1 | Ob-3 | HSL | Ploidy |
| Wc-2 | Tu-1 | Normal |  |
| Wc-2 | Wc-1 | Normal |  |
| Wei-0 | Be-1 | Normal |  |
| **Wei-1** | **REN11** | **Class 3 necrosis**; RC | |
| Wil-1 | Mir-0 | Normal |  |
| Wil-1 | Pla-0 | Normal |  |
| Wil-1 | Wil-3 | Normal |  |
| Wil-3 | Br-0 | Normal | RC |
| Wil-3 | Col-0 | Normal |  |
| Wl-0 | Bur-0 | Normal |  |
| Wt-1 | Wt-4 | Normal | RC |
| Wt-4 | Gr-3 | Normal |  |
| Wt-4 | Per-3 | Normal |  |
| Wt-4 | Wt-1 | Normal |  |
| Wt-5 | Rou-0 | Normal | RC |
| Yo-0 | Ba-1 | Normal |  |
| Yo-0 | Mr-0 | Normal |  |
| Zü-0 | Li-2 | Normal |  |
| Zü-0 | Sav-0 | Normal |  |
| Zü-1 | Be-1 | Normal |  |

Sha. =	Shahdara

RC =	Reciprocal cross;

HSL =	high seed lethality;

Ploidy =	Ploidy difference between parents.
