# Supplementary material for: Autoimmune Response as a Mechanism for a Dobzhansky-Muller-Type Incompatibility Syndrome in Plants
Source: PLoS Biol. 2007 Sep 4;5(9):e236. doi: 10.1371/journal.pbio.0050236 (PMC1964774; doi:10.1371/journal.pbio.0050236)
Supplement: Supplementary file 8 [file pbio.0050236.st005.doc]

**Table S5** Intercrossability of parents of several incompatibilities.

|  | Uk-1 | Uk-3 | Mir-0 | Se-0 | Bla-1 | Hh-0 | Shahdara | KZ10 | Mrk-0 |
| --- | --- | --- | --- | --- | --- | --- | --- | --- | --- |
| Uk-1 |  | I (III) | C | C | C | C | C | C | C |
| Uk-3 |  |  | C | C | C | C | C | C | C |
| Mir-0 |  |  |  | I (I) | C | C | C | C | C |
| Se-0 |  |  |  |  | C | I (I) | C | C | C |
| Bla-1 |  |  |  |  |  | I (II) | I (II) | C | C |
| Hh-0 |  |  |  |  |  |  | C | C | C |
| Shahdara |  |  |  |  |  |  |  | C | C |
| KZ10 |  |  |  |  |  |  |  |  | I (III) |
| Mrk-0 |  |  |  |  |  |  |  |  |  |

C = compatible, I = incompatible (“class” in parentheses).
