# Supplementary material for: Autoimmune Response as a Mechanism for a Dobzhansky-Muller-Type Incompatibility Syndrome in Plants
Source: PLoS Biol. 2007 Sep 4;5(9):e236. doi: 10.1371/journal.pbio.0050236 (PMC1964774; doi:10.1371/journal.pbio.0050236)
Supplement: Supplementary file 9 [file pbio.0050236.st006.doc]

**Table S6** *H. parasitica* resistance of hybrids and parents.

|  | **Cala2** | **Emco5** | **Noco2** | **Emwa1** |
| --- | --- | --- | --- | --- |
| Uk-1 | R | S | nd | nd |
| Uk-3 | R | S | nd | nd |
| Uk-1/Uk-3 | nd | R | nd | nd |
|  |  |  |  |  |
| Bla-1 | R | R | R | S |
| Hh-0 | R | R | R | S |
| Bla-1/Hh-0 | nd | nd | nd | S |
|  |  |  |  |  |
| Mrk-0 | S | S | nd | nd |
| KZ10 | S | R | nd | nd |
| Mrk-0/KZ10 | R | nd | nd | nd |
|  |  |  |  |  |
| Se-0 | S | R | S^1^ | S |
| Mir-0 | R | R | S^1^ | R |
| Se-0/Mir-0 | nd | nd | R | nd |

^1^weak sporulator (approx. 2 sporangiophores/cotyledon)
